# Disulfidptosis-Associated CCNB2: A Prognostic Biomarker and Immune Microenvironment Modulator in Prostate Cancer

**DOI:** 10.7150/jca.112791

**Published:** 2025-09-03

**Authors:** Wei Jiang, Qinghua Wang, Juan Zhou, Yan Zhao, Xin Qin, Xilei Li, Haopeng Li, Licheng Wang, Gang Wu

**Affiliations:** Tongji Hospital, Affiliated with Tongji University, Shanghai, China.

**Keywords:** Disulfidptosis, Prostate cancer, CCNB2, Prognosis

## Abstract

**Background** Disulfidptosis, a newly recognized form of cell death activated by disulfide bond stress, differs from apoptosis, ferroptosis, cuproptosis, and pyroptosis. Understanding its role in prostate cancer is essential for developing tailored therapeutic approaches for managing this condition. Here, we establish the first disulfidptosis-based molecular subtyping framework for prostate cancer (PCa) and identify CCNB2 as a novel regulator of disulfidptosis, revealing its dual role in apoptosis activation and immune microenvironment remodeling.

**Methods** We used consensus clustering to classify disulfidptosis into different subtypes and to study the unique characteristics linked to each one. We also developed a Dis score to measure the severity of each patient's subtype. We compared immune infiltration, pathway enrichment, and survival differences among the subtypes and revealed that the level of the score is significantly associated with the prognosis of PCa.Subsequently, we used Cytoscape software to further filter out hub genes and investigated how these genes influence the progression of PCa and their potential mechanisms through *in vitro* and *in vivo* experiments.

**Results** We identified three molecular subtypes associated with disulfidptosis (Cluster A, B, C) and three gene subtypes (GeneCluster A, B, C). Each subtype exhibited a distinct prognosis, level of immune cell infiltration, and biological pathway activation. Notably, Cluster B and GeneCluster B, characterized by elevated disulfidptosis gene expression, were correlated with favorable prognosis. Additionally, we discovered that patients with higher scores exhibited lower tumor mutational burden (TMB) and improved prognosis. Finally, our experimental results confirmed that downregulation of CCNB2 expression promoted disulfidptosis in prostate cancer cells, thereby inhibiting their migration and proliferation capacities.

**Conclusion** This study demonstrates that disulfidptosis can be utilized to stratify risk in patients with PCa. Furthermore, the CCNB2 gene emerges as a potential therapeutic target for prostate cancer by regulating disulfidptosis, thereby influencing the biological behaviors of PCa cells, including their proliferation and migration.

## Introduction

Prostate cancer (PCa), a prevalent malignancy among men, has experienced a notable increase in incidence over the past decade, coupled with a significant mortality rate 1. Despite ongoing advancements in treatment modalities, including surgery, radiotherapy, chemotherapy, and emerging targeted and immune therapies, managing PCa remains challenging. Particularly, when PCa progresses to advanced stages or develops resistance to castration therapy, patient prognosis becomes markedly poor 2. Recurrence of PCa considerably jeopardizes patient survival and prognosis 3, severely affecting quality of life and survival rates. This condition not only induces substantial physical and psychological distress but also imposes a significant economic burden 3. These challenges have driven the exploration of novel therapeutic mechanisms and targets.

In recent years, a research by Liu *et al.* 4 published in Nature Cell Biology has identified "disulfidptosis," a novel form of programmed cell death distinct from other recognized types, including apoptosis, necroptosis, cuproptosis, autophagy, ferroptosis, and pyroptosis 5. Disulfidptosis is driven by oxidative stress, wherein cancer cells—typically overexpressing solute carrier family 7 member 11 (SLC7A11)—enhance cystine uptake. In the absence of glucose and repair mechanisms, intracellular production of NADPH becomes insufficient or is excessively consumed, disrupting cellular redox homeostasis. This disruption prevents reduction of excess cystine to cysteine, resulting in the accumulation of cystine and the formation of disulfide bonds in actin cytoskeletal proteins. Consequently, the actin network collapses 6, ultimately inducing disulfidptosis.

Cyclin family members (CCNs) play pivotal roles in regulating cell cycle progression, each functioning at specific stages. Aberrant expression of these cyclins often leads to tumorigenesis, as demonstrated by CCND1 and CCNE1, whose abnormal expression enhances proliferation in lung adenocarcinoma 7, and cyclin A2, whose downregulation impairs colon cancer cell proliferation. Additionally, cyclin E1 has been identified as a potential target for ovarian cancer therapy 8. Moreover, Research has shown that inhibiting the cell cycle gene MELK can enhance the synergistic suppression of the cell cycle gene UBE2C, significantly impacting prostate cancer cell viability. Antibiotics like siomycin A strongly inhibit cell cycle genes linked to aggressive prostate cancer, inducing cell cycle arrest and offering a potential new treatment option 9. Collectively, these findings underscored the significance of cyclins in the development of various cancer types.

Cyclin B2 (CCNB2), a member of the B-type cyclin family, serves as a key regulator of the cell cycle 10. Previous research has demonstrated that CCNB2 is synthesized during the G1 phase and undergoes rapid downregulation during anaphase 11. Disruptions in CCNB2 functionality during the cell cycle result in the failure of the G2/M checkpoint, thereby promoting genetic mutations and initiating tumorigenesis 12. Furthermore, the role of CCNB2 in cancer progression and metastasis has been thoroughly investigated 13, 14. Aberrant expression of CCNB2 has been observed in various cancer types, including lung, bladder, and breast cancers 15, and elevated CCNB2 expression is associated with unfavorable prognosis in hepatocellular carcinoma (HCC) patients 16. In his latest research, Cai *et al.* investigated how circ_CCNB2 impacts the radiotherapy sensitivity of prostate cancer cells and its mechanism. The study found that circ_CCNB2 is upregulated in radioresistant prostate cancer tissues and cells. Knocking down circ_CCNB2 enhances the radiotherapy sensitivity of these cells, an effect achieved through autophagy inhibition. According to the search results, in the study by Cai *et al.*, it was shown that a circRNA encoded by Cyclin B2 (circ_CCNB2) is highly overexpressed in irradiation-resistant prostate cancer tissues and cells. Functionally, circ_CCNB2 can inhibit autophagy in prostate cancer cells by regulating genes targeted by the miR-30b-5p/KIF18A axis, thereby improving the sensitivity of radio-resistant prostate cancer to radiotherapy. This is in line with the content of the user's request 17. Although the influence of CCNB2 on cancer progression is well-documented, its potential involvement in PCa remains unexplored and warrants further investigation. To bridge this gap, our study: (i) Proposes disulfidptosis-driven subtypes for PCa risk stratification; (ii) Develops a Disulfidptosis Score (Dis score) integrating genomic and immune features; (iii) Validates CCNB2 as a therapeutic target through dual regulation of apoptosis and disulfidptosis - a mechanism previously unreported in PCa. Specifically, our research utilized bioinformatics methods to analyze the relationship between disulfidptosis and the prognosis and immune microenvironment of PCa, and further identified CCNB2 as a key hub gene associated with Disulfidptosis. Through *in vivo* and *in vitro* experiments, we confirmed that CCNB2 can modulate specific signaling pathways, thereby affecting the progression and prognosis of PCa. Consequently, CCNB2 may emerge as a novel therapeutic target for the future treatment of PCa.

## Materials and Methods

### Collection and processing of data

Information on somatic mutations and transcriptome data of PCa patients was obtained from the TCGA-PRAD (N = 495), CPGEA database (N = 137), and GEO database (GSE70768) (N = 220). The "SVA" package was utilized to adjust for batch effects within the TCGA-PCA, CPGEA, and GSE70768 datasets.

Inclusion Criteria:

Confirmed Diagnosis: Patients must have a definitive diagnosis of a specific tumor type based on pathological or cytological examination, in line with standard clinical diagnostic criteria. Normal samples must be confirmed as non-tumor tissue through pathological examination.

Sample Quality: Tumor samples should primarily be untreated primary tumors, with a minimum tumor cell content. For instance, TCGA mandates at least 80% tumor cell nuclei. Normal samples must also be morphologically intact, without significant lesions or necrosis.

Complete Clinical Information: Patient information such as age, gender, medical history, and treatment history should be available, as it helps link gene expression to clinical features.

Data Availability: Gene expression and sequencing data of patients should be complete and of high quality, without significant missing or abnormal values.

Exclusion Criteria:

Other Serious Illnesses: Patients with other serious diseases that might significantly impact the study gene expression or prognosis, such as severe cardiovascular or autoimmune diseases.

Treatment Interference: Patients who received gene expression - altering treatments like chemotherapy or radiotherapy before sample collection.

Sample Quality Issues: Samples with severe necrosis, contamination, or other quality issues that could compromise analysis accuracy.

Irrelevant to Research Objectives: Samples whose characteristics don't match the research objectives, e.g., studying a specific tumor stage but the sample is from a different stage.

### Identification of disulfidptosis subtypes and their correlation with biological pathways and TME

Disulfidptosis-associated genes were obtained from Liu *et al.* Using the ConsensusClusterPlus package, patients were classified into distinct disulfidptosis subtypes based on the expression profiles of these genes. The GSVA and ssGSEA algorithms were then applied to investigate differences in biological functions across the subtypes. Additionally, the ssGSEA method was employed to evaluate immune cell infiltration in PCa patients.

### Building a disulfidptosis-related risk score

The 'limma' package was used to identify differentially expressed genes (DEGs) across various clusters, defining DEGs as those with an absolute log₂ fold change greater than 1 and an adjusted *p*-value below 0.05. Prognosis-related DEGs were identified through univariate Cox regression analysis. Subsequently, Lasso Cox regression was applied to construct a disulfidptosis-related risk score, calculated by multiplying the expression levels of each gene by its respective coefficient for each sample. Finally, Kaplan-Meier (KM) survival analysis was conducted to evaluate differences in overall survival (OS) 18.

### Cell culture and transfection

PCa cell lines DU145, C4-2, PC3, and the human prostate hyperplasia cell line RWPE-1 were obtained from the Chinese Academy of Sciences Cell Bank. These cells were cultured in RPMI 1640 medium supplemented with 10% fetal bovine serum (FBS) at 37°C with 5% CO₂. Following the manufacturer's instructions, cells were transfected with si-CCNB2 using Lipofectamine 2000 (Invitrogen, USA). For transfection, si-CCNB2 and Lipofectamine 2000 were diluted in serum-free Opti-MEM medium at a 1:1 ratio, mixed, and incubated on ice for 15 mins before being added to the cells 19. siRNA fragments were sourced from Guangzhou RiboBio Co., Ltd.

The sequences of the siRNA are as follows:

si-CCNB2-1 forward (CCAGUGAUUUGGAGAAUAUTT), reverse (AUAUUCUCCAAAUCACUGGTT), si-CCNB2-2 forward (GGCCAAGAAUGUGGUGAAATT), reverse (UUUCACCACAUUCUUGGCCTT).

### Real-time qPCR

RNA was extracted from the cell lines using the TRIzol kit (Invitrogen, USA) according to the manufacturer's instructions. The PrimeScript RT kit (Takara, Japan) was utilized to synthesize cDNA from the extracted RNA. For quantitative PCR (qPCR) analysis, a 10 µL reaction system was prepared with cDNA and gene-specific primers following the TAKARA kit instructions, and real-time qPCR was performed 20.

The sequences used in RT-qPCR are as follows:

CCNB2 forward (CCGACGGTGTCCAGTGATTT), reverse (TGTTGTTTTGGTGGGTTGAACT), GAPDH forward (GUAUGACAACAGCCUCAAGTT), reverse (CUUGAGGCUGUUGUCAUACTT).

### Western blot

Total protein was extracted from PCa cells using RIPA lysis buffer. Protein concentrations were determined using the Beyotime (Shanghai) protein concentration determination kit, following the manufacturer's protocol. The BCA working solution was prepared based on the number of samples and added to both the protein samples and standards. After incubating for 30 mins at room temperature, absorbance at 562 nm was measured using a microplate reader (Varioskan LUX, USA). The BCA method was employed to determine the protein concentration of the samples. To facilitate protein loading, a 4:1 ratio of protein loading buffer was added to the samples, and the mixture was incubated for 15 minutes at 100°C 21.

For electrophoresis, proteins were separated using a 10% SDS-PAGE gel and subsequently transferred to a PVDF membrane. The membrane was blocked with a 5% BSA solution at room temperature for 1 hour. Following this, the membrane was incubated overnight at 4°C with various antibodies, including CCNB2 antibody (Proteintech, China), GAPDH antibody (Proteintech, China), Caspase-3 antibody (Proteintech, China), BCL-2 antibody (Abmart, China), BAX antibody (Proteintech, China), and SLC7A11 antibody (Abmart, China), among others 22.

### CCK-8 assay

PC3 and DU145 cells, both untransfected and transfected, were seeded in a 96-well plate at a density of 5,000 cells per well. In each well, 10 μL of CCK-8 solution (Uelandy, China) and 100 μL of 10% culture medium were added at time intervals of 0 hour, 24 hours, 48 hours, and 72 hours post cell adhesion. After incubation at 37°C for 2 hours, the absorbance at 450 nm was measured using a microplate reader (Varioskan LUX, USA).

### Wound healing assay

Cells post-transfection were plated in a 6-well plate at an optimal density. Upon reaching confluence, a 1000 μl pipette tip was used to create scratches in the cell monolayer. In order to quantify the wound healing process, the wound area was measured every 24 hours using Image J. For this purpose, the healing rate was calculated as follows: (original wound area - unhealed wound area)/original wound area * 100% 23.

### Transwell assay

Cells were seeded in the upper chamber of a transwell insert with serum-free medium at an appropriate density, while the lower chamber was filled with RPMI 1640 medium supplemented with 10% fetal bovine serum (FBS). After a 24-hour incubation period, cells that had migrated to the lower surface of the chamber were fixed with 4% paraformaldehyde and stained with crystal violet 24.

### EDU assay

Following transfection, cells were plated in 96-well plates at a density of 1 × 10^4^ cells per well and incubated for 24 hours. The EDU solution was prepared by diluting the stock solution in EDU medium to a final concentration of 50 μM. Subsequently, 100 μL of the diluted EDU medium was added to each well and incubated for an additional 2 hours. The cells were then fixed, and 100 μL of Click-iT working solution (Uelandy, China) was applied for staining. DNA counterstaining was performed, and the cells were visualized under a fluorescence microscope 25.

### Clonogenic assay

Eight hundred transfected cells were uniformly seeded into each well of a 6-well plate and incubated for 10 days. After the incubation period, cells were washed twice with PBS, fixed with 4% paraformaldehyde, and stained with crystal violet. The number of cell colonies formed was subsequently counted.

### Immunofluorescence

Post-transfection cells were evenly plated in a 6-well plate at an appropriate density. Cells were then fixed with 4% paraformaldehyde and permeabilized with a 0.2% Triton X-100 solution at room temperature. Following permeabilization, cells were incubated with 200 μL of phalloidin working solution for 20 mins and counterstained with DAPI. The distribution of the CCNB2 protein was observed using immunofluorescence microscopy.

### *In vivo* experiments

To establish a reliable *in vivo* model, twelve 4-week-old sex-matched BALB/c mice were obtained from Shanghai Shengchang Biotechnology Co., Ltd and randomly divided into two groups: Sh-Control and Sh-CCNB2. These mice were maintained in a specific pathogen-free (SPF) environment at the experimental animal facility of Tongji Hospital, Tongji University. For tumor modeling, twelve nude mice were subcutaneously injected with tumor cells from both groups. Tumor growth was monitored every five days over the course of one month. Humane euthanasia was performed on the mice at the end of the experiment, and the tumors were dissected, photographed, and weighed. Tumor samples were subsequently stored in liquid nitrogen or fixed in formaldehyde for future experiments.

### Statistical analysis

We performed bioinformatics analyses using R version 4.1.3. Continuous variables were tested for normality with the single-sample Kolmogorov-Smirnov test. If normally distributed, we proceeded with t-tests or one-way ANOVA for data analysis. These experiments were independently repeated three times, and statistical significance was determined by a *P-*value of less than 0.05.

## Results

### Characteristics of disulfidptosis related genes

**Figure 1:**
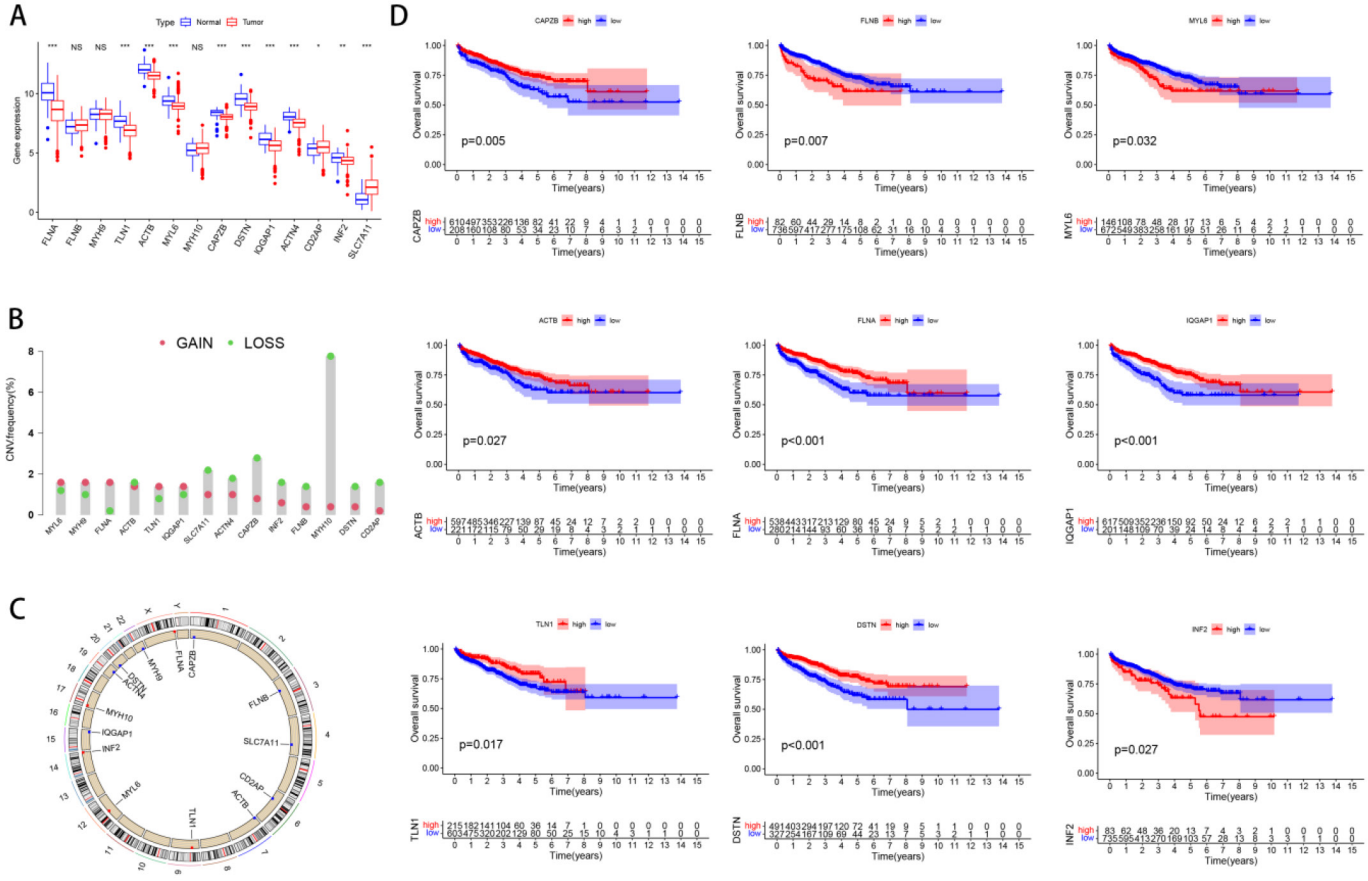
** Characteristics of disulfidptosis related genes.** (A) The distribution of DEGs' expression between PCa and normal tissues. (B) The occurrence of copy number variations (CNVs) in genes linked to disulfidptosis. (C) The chromosomal locations of CNVs in Dis genes. (D) The correlation between Dis genes and the prognosis of PCa.

Firstly, we analyzed DEGs' expression, copy number variations (CNVs) and the correlation between these genes and the prognosis of PCa. Most of these genes were upregulated in PCa (Figure 1A). In addition, we found that MYH10 had the highest gene mutation frequency (Figure 1B). The amplification and deletion of CNV were shown as follows (Figure 1C). Among these genes, high expression of certain genes, such as FLNA and DSTN, is positively correlated with the prognosis of prostate cancer patients, while elevated expression of other genes, including FLNB and INF2, is associated with a negative prognosis (Figure 1D).

### The relevant characteristics of three clusters

**Figure 2:**
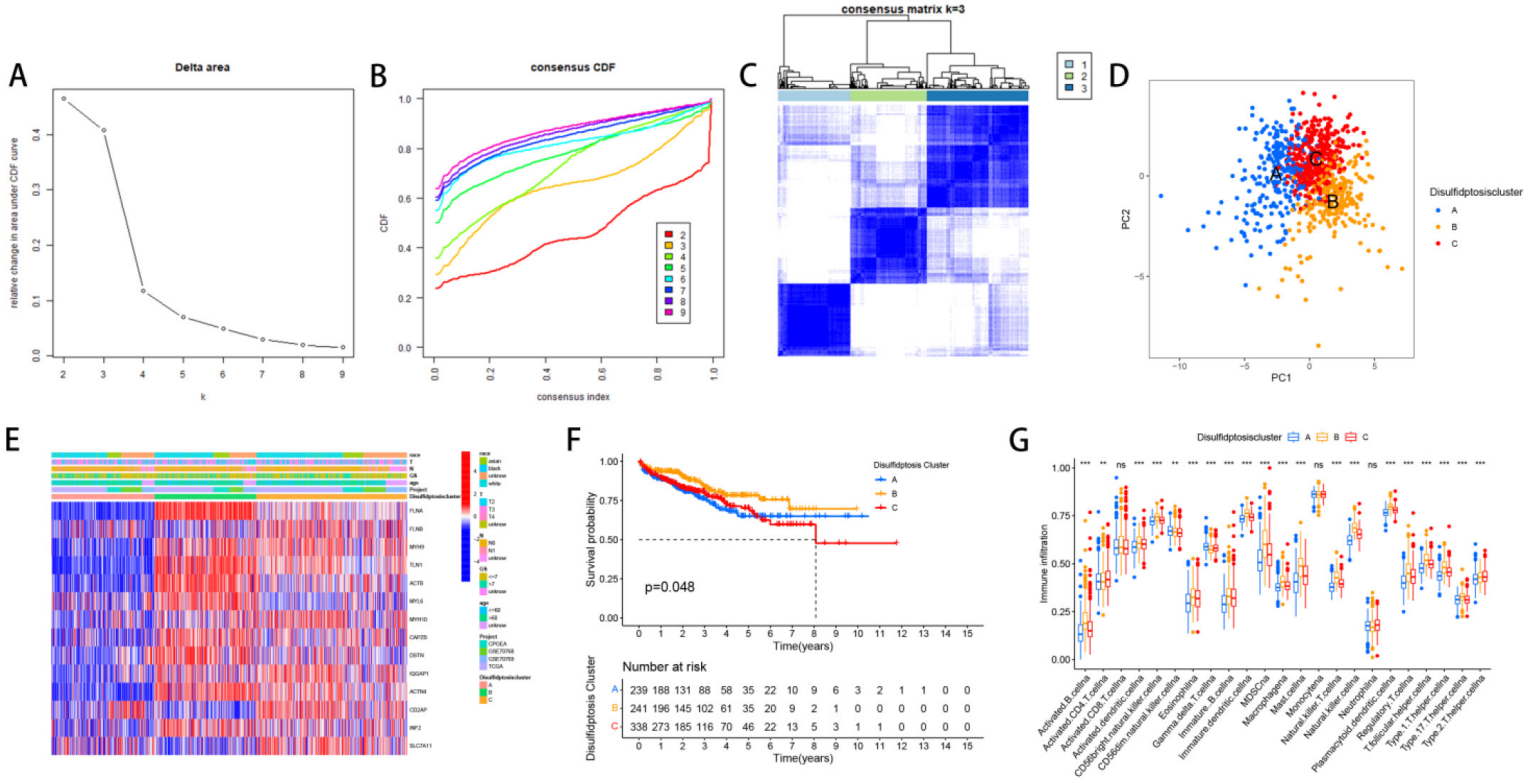
** The relevant characteristics of three Clusters.** (A-C) A heatmap of the consensus matrix delineates the three Clusters (k = 3) and their respective associated regions. (D) Analysis of PCa revealed significant differences in the transcriptome among the three subgroups. (E) Variations in clinical characteristics and Dis gene expression were observed among the distinct clusters. (F) KM curve analysis was conducted to evaluate the variations in overall survival (OS) across the various clusters (G) Analysis of immune cell infiltration levels using ssGSEA within clusters.

We divided PCa patients into three subtypes and analyzed characteristics of these Clusters such as overall survival and immune infiltration. Using a consensus clustering algorithm, PCa patients were divided into three subtypes, namely Discluster A, B, and C (Figure 2A, B, C). PCa analysis revealed significant differences among the three subtypes (Figure 2D) and significant differences in the expression of Dis genes among the three subtypes (Figure 2E). Among them, Cluster B, which had higher Dis gene expression, had a better survival prognosis (Figure 2F). The ssGSEA analysis revealed notable variations in immune infiltration levels between the two subtypes, with significant increases in Cluster B for Immature dendritic cells, Activated B cells, Activated dendritic cells, MDSCs, Mast cells, Regulatory T cells, Eosinophils, Immature B cells, Plasmacytoid dendritic cells, Macrophages, T follicular helper cells, Type 1 T helper cells, Natural killer T cells, Natural killer cells, and Type 17 T helper cell infiltration (Figure 2G).

### Discluster-related DEGs and their biological functions

**Figure 3:**
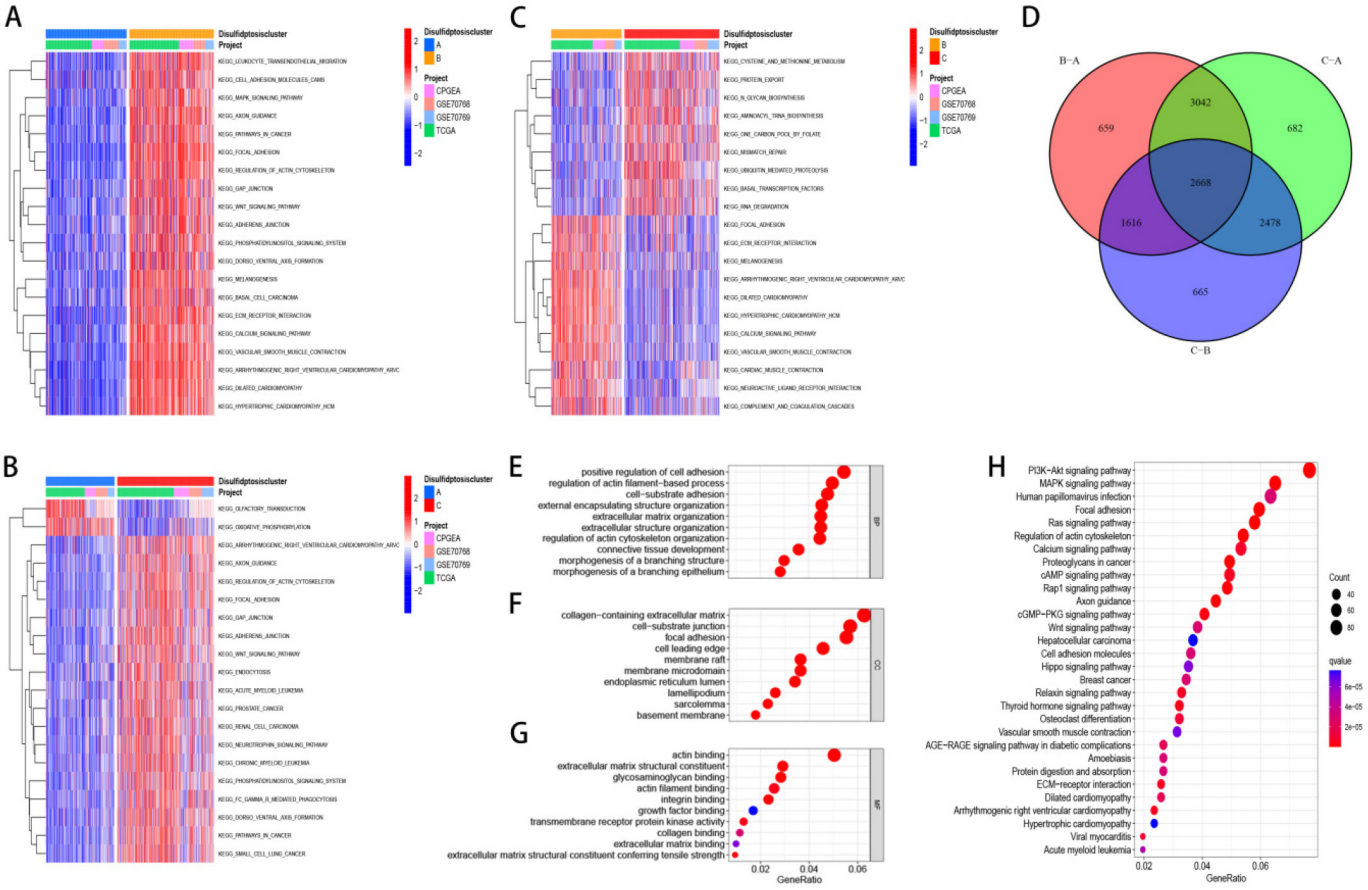
** Discluster-related DEGs and their biological functions.** (A-C) GSVA analysis between subtypes. (D) The venn figure showed 2668 DEGs between the three Clusters. (E-G) Utilized GO for enrichment analysis of genes that were differentially expressed. (H) Utilized KEGG for enrichment analysis of genes that were differentially expressed.

We selected 2668 DEGs between the three clusters and Utilized GO and KEGG for enrichment analysis of these genes. Furthermore, we examined the distinctions in biological pathways and immune infiltration between the two subtypes. GSVA results showed that Discluster B was enriched in pathways such as cell adhesion molecules, WNT signaling pathway, MAPK signaling pathway, ECM-receptor interaction, pathways in cancer and so on (Figure 3A-C). Using the "Limma" package, 2668 DEGs related to disulfide downregulation were identified (Figure 3D). GO analyses showed that these DEGs were predominantly linked to the positive regulation of adhesion, regulation of collagen-containing extracellular matrix, focal adhesion (CC), actin binding (MF), and actin filament-based processes (BP) (Figure 3E, F, G). KEGG analyses showed that the related signaling pathways were the focal adhesion, MAPK signaling pathways, and PI3K-Akt pathways (Figure 3H).

### The relevant characteristics of three GeneClusters

**Figure 4:**
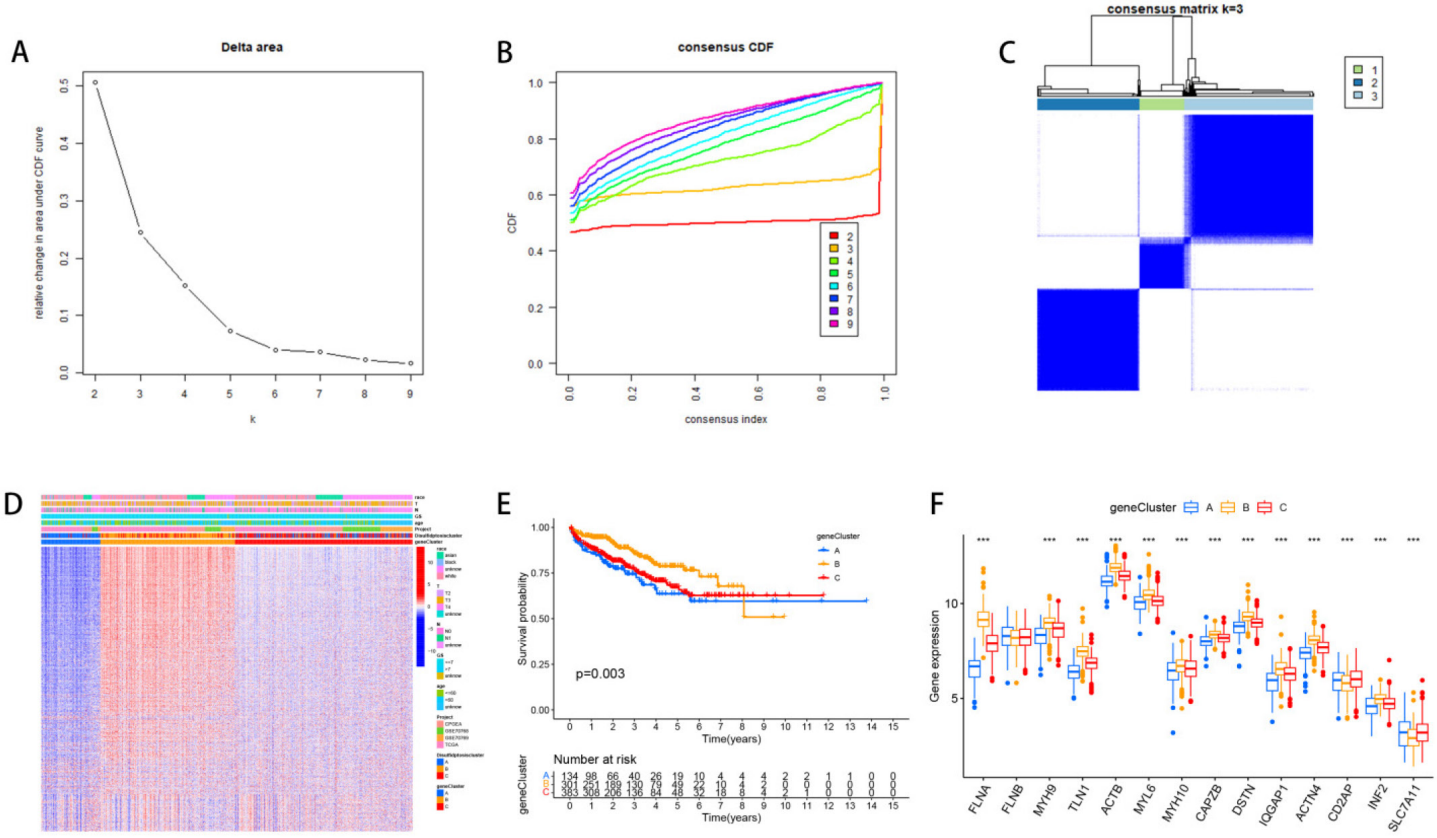
** The relevant characteristics of three GeneClusters.** (A-C) Consensus matrix heatmaps define three GeneClusters (k = 3) and their associated regions. (D) Variations in clinical characteristics and expression levels of Dis genes across distinct GeneClusters were observed. (E) To evaluate differences in overall survival, a Kaplan-Meier (KM) curve analysis was performed. (F) We analyzed the differential expression of Dis genes across the three GeneClusters.

Based on 2668 DEGs, we divided PCa patients into three GeneClusters and analyzed characteristics of these Clusters such as overall survival and immune infiltration. Using consensus clustering analysis, three genetic subtypes of patients were identified based on the expression patterns of the DEGs (Figure 4A, B, C). It was found that genetic subtype B exhibited high expression levels of these DEGs (Figure 4D), and subtype B had the best survival prognosis (Figure 4E). Analysis of the expression of disulfide genes in the three genetic subtypes revealed that TLN1, FLNA, ACTB, MYH9, MYL6, DSTN, MYH10, IQGAP1, ACTN4, and CAPZB were highly expressed in gene cluster B (Figure 4F).

### The relationships between the Dis score and the clusters, TMB, immune infiltration and prognosis

**Figure 5:**
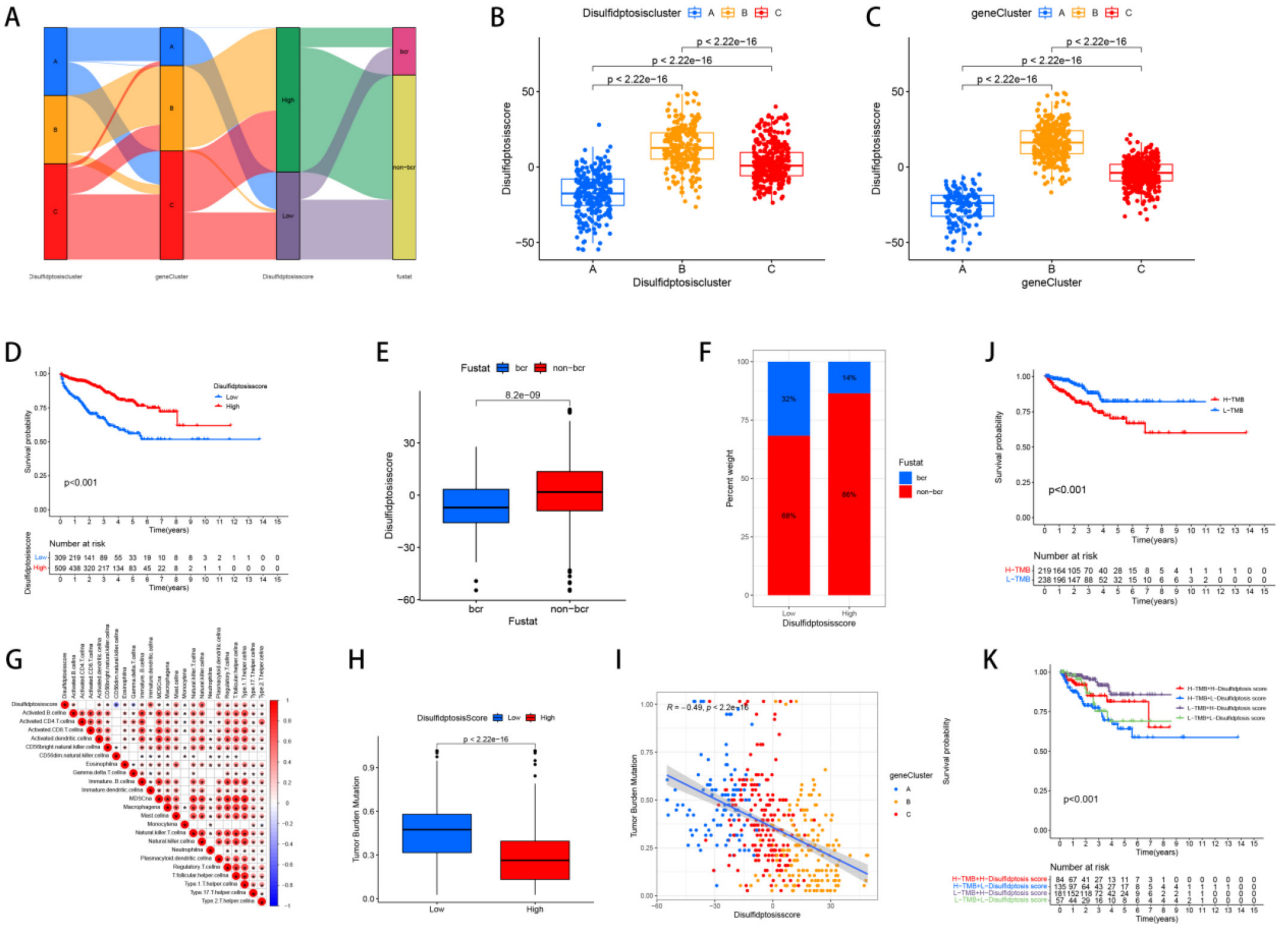
** The relationships between the Dis score and the clusters, TMB, immune infiltration and prognosis.** (A) Sankey diagrams revealed the correlation between the Dis score and subtypes, genetic subtypes, and prognosis. (B) Boxplots revealed the relationship between the Dis score and subtypes. (C) Boxplots revealed the relationship between the Dis score and genetic subtypes. (D) KM curves revealed the survival differences between high and low score groups. (E) Boxplots revealed the relationship between the Dis score and the rate of biochemical recurrence in patients with PCa. (F) Bar charts revealed the relationship between the Dis score and the rate of biochemical recurrence in patients with PCa. (G) The correlation between the Dis score and the infiltration of immune cells. (H) Box plots revealed the correlation between the Dis score and TMB. (I) Regression curves indicated a negative correlation between the Dis score and TMB. (J) KM survival curves revealed the relationship between the prognosis of PCa patients and TMB. (K) KM survival curves revealed the relationship between the prognosis of PCa patients and TMB as well as the Dis score.

Fourteen genes were subsequently identified through the application of LASSO and multivariate Cox regression analyses: SLC7A11, FLNA, CD2AP, FLNB, ACTB, ACTN4, INF2, MYH9, TLN1, MYL6, IQGAP1, MYH10, DSTN, and CAPZB. Based on these genes, we established the Disulfide Score (Dis score) which was calculated as follows: Disulfide Score=∑ (Expression Level of Genei×Coefficienti). Then, we revealed the relationships between the Dis score and the clusters, TMB, immune infiltration and prognosis. Sankey and box plots illustrate the relationship between the Dis score and molecular subtypes, genetic subtypes (Figure 5A, B, C). Overall, Cluster B and Gene cluster B had higher Dis scores. Our KM survival curves further indicated that patients with higher Dis scores had better survival prognoses (Figure 5D) and corresponded to higher non-biochemical recurrence rates (Figure 5E, F).

Upon analyzing the correlation between the Dis score and immune cell infiltration, we determined that patients with elevated scores exhibit more pronounced immune infiltration of MDSCs, Macrophages, Natural killer T cells, Activated B cells, T follicular helper cells, CD56 bright Natural killer cells, Monocytes, Mast cells, Type 1 T helper cells, Regulatory T cells, Type 2 T helper cells, Type 17 T helper cells, Eosinophils, Immature B cells, Plasmacytoid dendritic cells, Activated dendritic cells, Immature dendritic cells, and Natural killer cells (Figure 5G). The study results showed that the TMB of the high-risk cohort was significantly reduced, and there was a negative correlation between the two (Figure 5H, I). In addition, it was observed that patients with lower TMB had better prognoses (Figure 5J), and high-risk group patients with low TMB had a significant survival advantage (Figure 5K).

### The approach and steps in selecting experimental gene (CCNB2)

**Figure 6:**
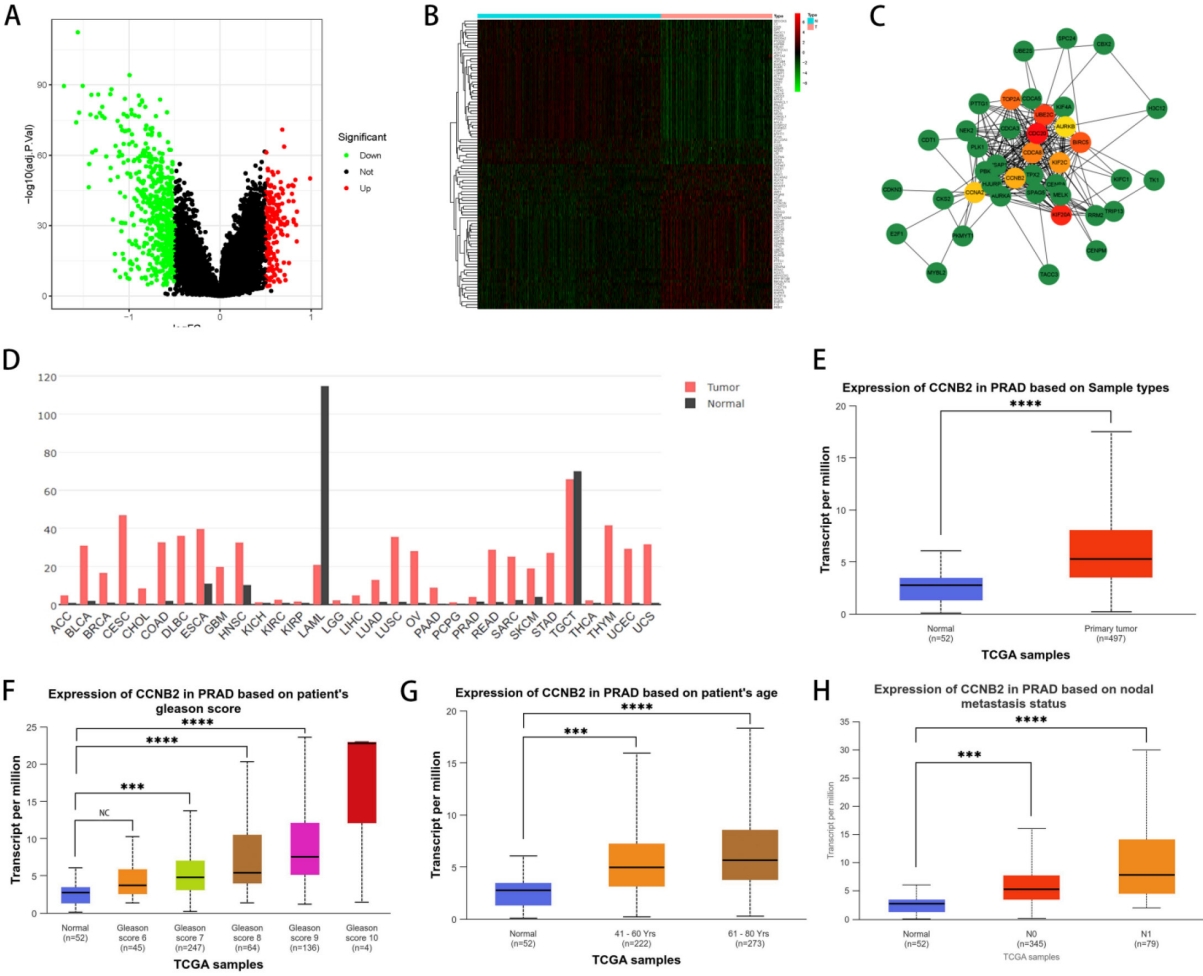
** The approach and steps in selecting experimental gene (CCNB2).** (A) Differentially expressed genes were identified between groups with high and low scores, with green representing downregulated genes in the high-scoring group and red representing upregulated genes in the high-scoring group. (B) A heatmap revealed the specific genes that are differentially expressed. (C) The top 10 hub genes were identified by constructing a protein-protein interaction (PPI) network with Cytoscape software. (D) The expression of CCNB2 in various tumor tissues as per the TCGA database. (E) The expression of CCNB2 in PCa as per the TCGA database. (F) The Gleason score of PCa was positively associated with CCNB2 expression. (G) CCNB2 expression showed a positive correlation with the age of PCa patients. (H) Lymph node metastasis in PCa was positively associated with CCNB2 expression (***P < 0.05, ****P < 0.01).

To delve into the relationship between disulfidptosis and the progression of PCa, we selected CCNB2 as our hubgene for in-depth experimental validation. We employed an algorithm to detect genes exhibiting differential expression between patients with high and low scores, as illustrated in the figure, which included both upregulated and downregulated genes (Figure 6A, B). Based on these differentially expressed genes, we utilized the STRING database and Cytoscape software to construct a protein-protein interaction (PPI) network from which we selected the top 10 hub genes (Figure 6C). Subsequently, we further explored the functions of these genes. Among them, CCNB2, which encodes a cyclin protein, is a member of the cyclin family and primarily functions during the G2/M transition phase, being upregulated in human cancers 26, 27. For instance, overexpression of CCNB2 is associated with poor prognosis in hepatocellular carcinoma (HCC), and reduced expression of this gene can suppress the metastasis and invasion of bladder cancer. Ni et al. proposed that CCNB2 may serve as a potential biomarker as well as a therapeutic target for lung adenocarcinoma (LUAD) 28. These discoveries suggest that targeting CCNB2 could represent a new approach to treating cancer. Further analysis of the TCGA database indicated that CCNB2 exhibits high levels of expression across a spectrum of cancers, including PCa (Figure 6D, E) and the elevated level of its expression was positively correlated with Gleason score, age, and lymph node metastasis in PCa patients (Figure 6F, G, H), suggesting that the CCNB2 gene was a factor contributing to risk for PCa. However, previous reports have not elucidated how CCNB2 functions in PCa and its underlying mechanisms. Therefore, we selected CCNB2 as a hub gene for in-depth experimental validation.

### The selected experimental cell lines and the knockdown effect of the CCNB2 gene

**Figure 7:**
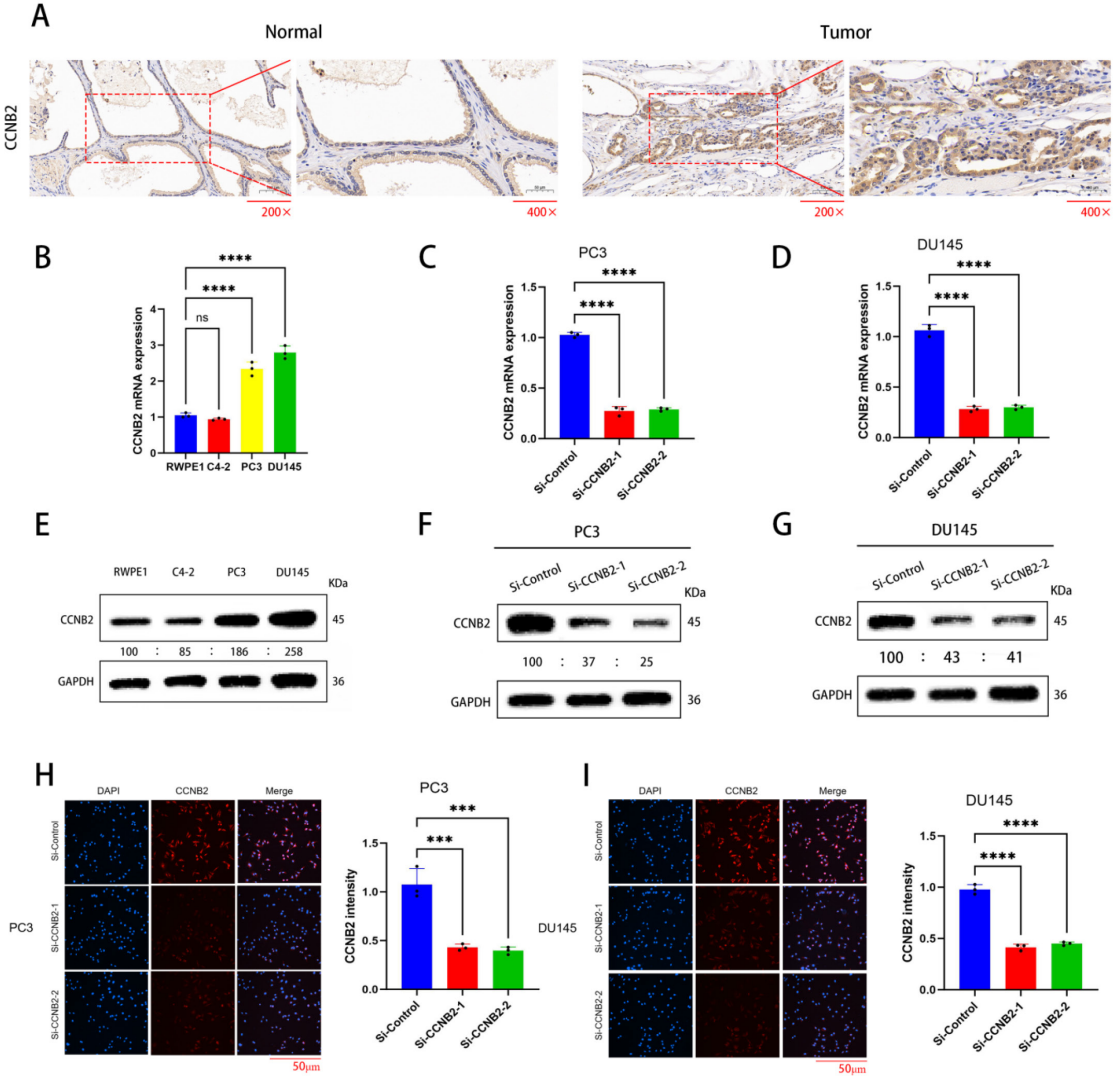
** The selected experimental cell lines and the knockdown effect of the CCNB2 gene.** (A)Immunohistochemistry shows a difference in CCNB2 expression between tumor tissue and normal tissue. (B) mRNA expression of CCNB2 in PCa cell lines. The experiments were conducted three times and analyzed Utilizing a one-way ANOVA test with a statistical significance threshold of *P* < 0.05. (C) Knockdown of CCNB2 expression in PC3 cells using siRNA, and verification of efficiency by qPCR.(D) Knockdown of CCNB2 expression in DU145 cells using siRNA, and verification of efficiency by qPCR. (E) Protein expression of CCNB2 in PCa cell lines. The experiments were conducted three times and analyzed Utilizing a one-way ANOVA test with a statistical significance threshold of* P* < 0.05. (F) Knockdown of CCNB2 expression in PC3 cells using siRNA, and verification of efficiency by WB. (G) Knockdown of CCNB2 expression in DU145 cells using siRNA, and verification of efficiency by WB. (H) Knockdown of CCNB2 expression in PC3 cells using siRNA, and verification of protein expression by immunofluorescence. (I) Knockdown of CCNB2 expression in DU145 cells using siRNA, and verification of protein expression by immunofluorescence (***P < 0.05, ****P < 0.01).

Through immunohistochemistry, we demonstrated that there was indeed a difference in CCNB2 expression between prostate cancer tissue and benign prostatic hyperplasia (Figure 7A). Then, we analyzed the expression of the CCNB2 gene in PCa and benign prostatic tissues using western blot and qPCR: Compared to RWPE-1 (benign prostatic hyperplasia tissue), the expression levels of CCNB2 in PC3 and DU145 were higher in PCa tissues, while C4-2 exhibited lower expression levels in tumors (Figure 7B, E). Therefore, we selected PC3 and DU145 as experimental cell lines. Subsequently, we knocked down CCNB2 in PC3 and DU145 cell lines using siRNA and confirmed the knockdown efficiency with qPCR, WB and immunofluorescence (Figure 7C, D, F, G, H, I). Simultaneously, immunohistochemistry revealed a significant downregulation of CCNB2 expression in PC3 cells where CCNB2 was knocked down.

### *In vitro* experimental verification of CCNB2 function

**Figure 8:**
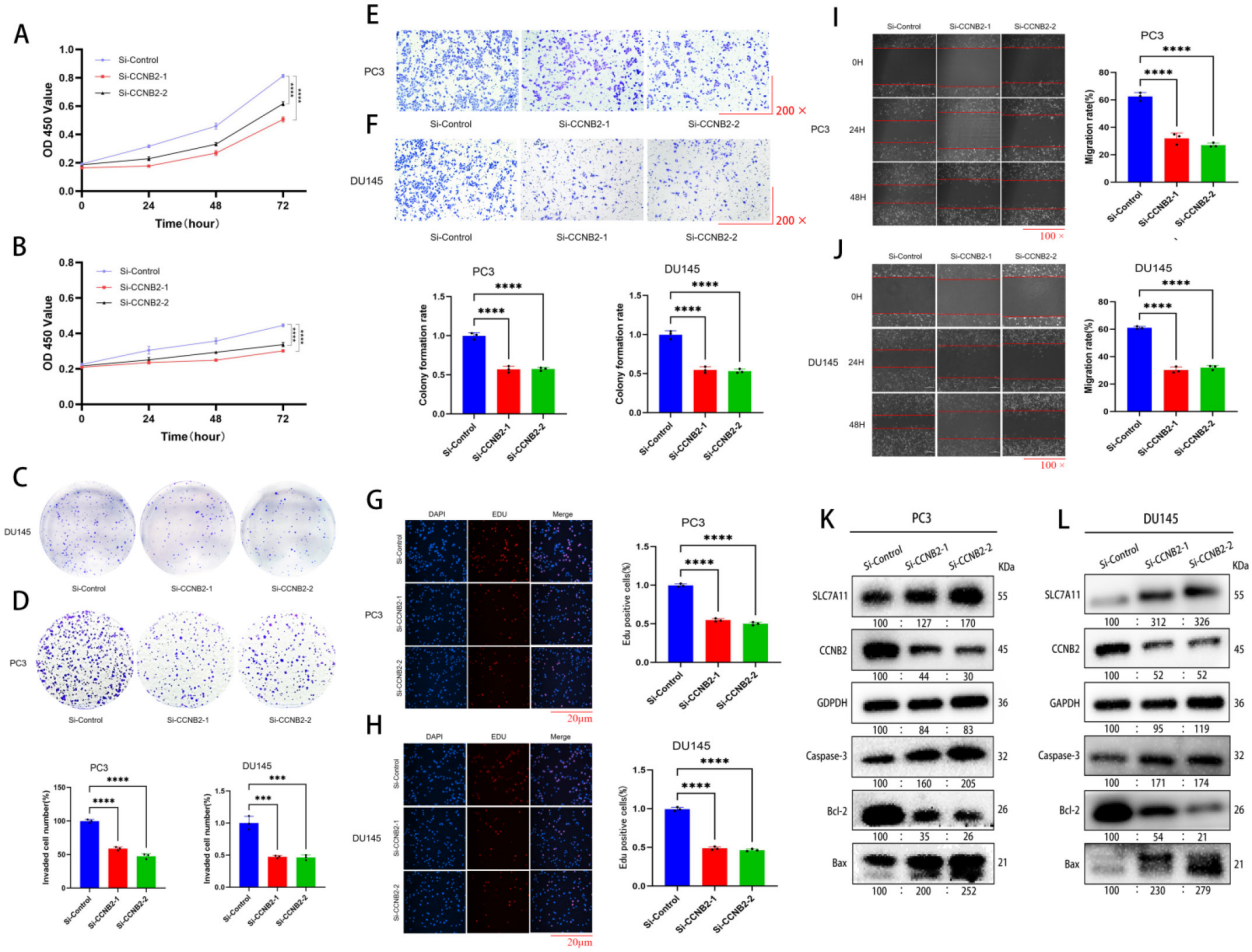
***In vitro* experimental verification of CCNB2 function.** (A(CCK8), D(colony formation), G(EDU)) These assays showed a significant reduction in the growth of PC3 cells following the knockout of CCNB2, with the CCK8 data depicted as a line chart and the remaining results as bar graphs. (B(CCK8), C(colony formation), H(EDU) These assays showed that the proliferation capacity of DU145 cells was diminished following the knockout of CCNB2. (E) The Transwell assay indicated that CCNB2 knockdown in PC3 cells led to a decreased invasive ability. (F) Similarly, the assay showed a reduction in the invasive capacity of DU145 cells post-CCNB2 knockdown. (I) The scratch assay revealed that knocking down CCNB2 in PC3 cells resulted in a reduced ability to migrate. (J) The scratch assay indicated that the migratory capacity of DU145 cells was reduced upon CCNB2 knockdown. (K) The levels of protein expression related to disulfidptosis and apoptosis pathways were observed to change in the PC3 cell line following CCNB2 knockdown. (L) The levels of protein expression related to disulfidptosis and apoptosis pathways were altered in the DU145 cell line upon CCNB2 knockdown (***P < 0.05, ****P < 0.01).

By CCK-8, colony formation, EDU, scratch, and Transwell invasion assays, we indicated that the rate of growth in PC3 and DU145 cells was notably changed after downregulation of CCNB2 expression. Cell proliferation experiments using CCK-8, colony formation, and EDU assays indicated that the rate of growth in PC3 and DU145 cells was notably reduced after downregulation of CCNB2 expression (Figure 8A, B, C, D, G, H). Additionally, Transwell invasion assays demonstrated a decrease in invasive capacity after knockdown (Figure 8E, F). Furthermore, scratch assays showed that the migratory ability of PC3 and DU145 cells was notably inhibited after CCNB2 downregulation (Figure 8I, J). To explore the mechanisms by which CCNB2 affects the biological behavior of PCa, we performed CCNB2 gene knockdown in PC3 and DU145, and then analyzed the impact of this knockdown on the apoptotic pathway (Caspase-3, Bcl-2, Bax) and the disulfidptosis pathway (SLC7A11) using western blot (Figure 8K, L). We found that the expression of apoptotic and disulfidptosis signaling molecules was enhanced after CCNB2 knockdown, indicating that these two pathways were activated. This indirectly suggests that CCNB2 may play a role in the disulfidptosis mechanism of PCa.

### *In vivo* experimental verification of CCNB2 function

**Figure 9:**
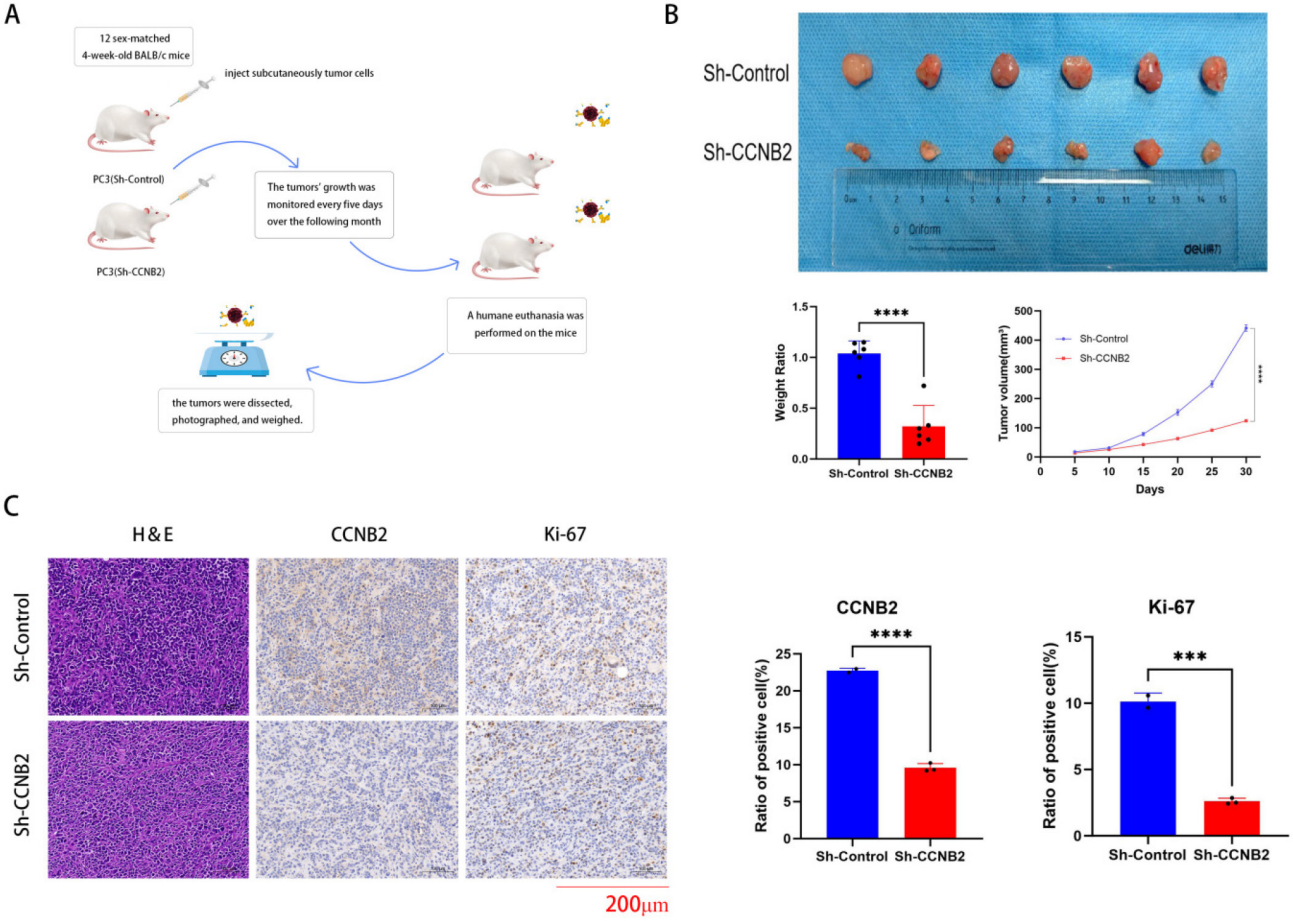
***In vivo* experimental verification of CCNB2 function.** This flowchart briefly described the handling of mice and tumors in our *in vivo* experimental process. (B) Silencing CCNB2 suppresses tumor growth. To investigate this, PC3 cells with either a vector control or silenced CCNB2 were injected subcutaneously into mice, which were euthanized three weeks post-injection. Tumor volumes were then calculated employing the formula: (length × width^2) × 0.5. (C) CCNB2 and ki67 immunohistochemical staining of the tumors, as detailed in the legend for panel A, was shown at a magnification of ×200, with scale bars representing 200 μm (***P < 0.05, ****P < 0.01).

We conduct analysis with immunohistochemistry to verify the knockdown efficiency of CCNB2 and *in vivo* experiments to prove that the knockdown of CCNB2 can indeed influence the progression of PCa once again. Ultimately, to substantiate the hypothesis that CCNB2 promotion contributes to the progression of PCa, an *in vivo* mouse model was employed to investigate the impacts of the CCNB2 gene on tumorigenesis. The study found that PC3 cells with decreased CCNB2 expression substantially suppressed tumor growth (Figure 9B), accompanied by a notable decrease in ki67, a marker indicative of tumor proliferation, thus preliminarily suggesting that the knockdown of CCNB2 inhibits cellular proliferative activity (Figure 9C). And the flowchart of the *in vivo* experiment is as follows (Figure 9A).

## Discussion

Disulfidptosis, a recently identified type of cell death, is governed by distinct molecular mechanisms. It has a strong correlation with numerous diseases and tumors, with cancer being characterized by resistance to cellular demise and uncontrolled proliferation. Recently, extensive research has been conducted on various forms of cell death, such as ferroptosis, apoptosis, pyroptosis, and necroptosis 29. Previous researchers uncovered a fresh type of cell death known as disulfidptosis, presenting new avenues for cancer therapy. We stratified PCa into three subtypes based on disulfidptosis characteristics. We observed that most DEGs were mutated in PCa tissues, further indicating the differential expression of DEGs in cancer tissues. Among them, ACTB, DSTN, IQGAP1, FLNA, CAPZB, and TLN1 were protective factors for PCa prognosis, while MYL6, FLNB, and INF2 were risk factors for PCa prognosis. Our study further elucidated the influence of DEGs on the outcome of PCa.

Our research highlighted notable variations in the pathways and functions among the three subtypes of disulfidptosis, along with distinct differences in KEGG and GO annotations, primarily concentrated in areas such as WNT signaling, ECM-receptor interaction, cancer-related pathways, MAPK signaling, cell adhesion molecules and so on. These pathways and functions may hold implications for PCa prognosis. Our current investigation also uncovered variations in immune cell infiltration across the three disulfidptosis subtypes. In essence, our studies suggested that DRGs impacted multiple facets of PCa patients, encompassing various signaling pathways and immune cell infiltration, influencing PCa patient survival or prognosis. However, the precise mechanisms and roles involved await further exploration. The three subtypes showed significant differences in prognosis and immune infiltration. Discluster B and GeneCluster B were characterized by high expression of disulfidptosis, good prognosis, and abundant immune cell infiltration. Therefore, this stratification of PCa is beneficial for personalized cancer treatment.

To comprehensively stratify the disulfidptosis characteristics of PCa patients, we developed a score specifically for disulfidptosis. The Dis score was calculated based on 14 essential characteristic genes: SLC7A11, FLNA, CD2AP, FLNB, ACTB, ACTN4, INF2, MYH9, TLN1, MYL6, IQGAP1, MYH10, DSTN, and CAPZB. The high-scoring group was characterized by good prognosis, low tumor mutational burden (TMB), and abundant immune cell infiltration. The low-scoring group showed opposite characteristics. Therefore, the risk score we had developed could assist in evaluating the prognosis of patients with PCa and identify those who may benefit from immunotherapy. This will aid in the more targeted use of pharmacological treatments, potentially leading to improved patient outcomes.

In our further investigation, vitro experiments were conducted to investigate the influence of the CCNB2 on the progression of PCa. As a significant member of the cyclin family, CCNB2 plays a pivotal role in regulating cell cycle progression and proliferation. Cyclins B1 and B2, belonging to the cyclin family, play a crucial role in regulating the cell cycle through their interactions with other cell cycle control molecules 30, 31. They associate with CDK1 to facilitate mitosis, especially during the phase transition from G2 to M in the cell cycle 32, 33. Cai et al. found that circ_CCNB2 is overexpressed in radioresistant prostate cancer tissues and cells. When circ_CCNB2 is knocked down, it suppresses the colony formation and metastatic ability of radioresistant prostate cancer cells while promoting apoptosis. This indicates that knocking down circ_CCNB2 enhances the radiotherapy sensitivity of prostate cancer cells, thereby slowing tumor progression 17. However, previous studies on CCNB2 in PCa did not involve its relationship with the disulfidptosis pathway. Notably, our discovery that CCNB2 knockdown triggers disulfidptosis, redefining its functional paradigm. While CCNB2 is canonically associated with G2/M progression, this cell cycle-independent mechanism explains its broader impact on PCa progression and therapy resistance - a dimension overlooked in prior oncology studies. We had demonstrated through CCK8, scratch, and transwell invasion assays that the knockdown of CCNB2 inhibited the proliferation, migration, and invasion capabilities of prostate cancer. Additionally, WB confirmed that the suppressive effects of CCNB2 on the biological behaviors of PCa may stem from its regulation of apoptotic and disfidptosis signaling pathways. Furthermore, our animal experiments have further illustrated that the knockdown of CCNB2 can inhibit the growth of PCa in mice. These results indicated that CCNB2 held potential as a promising therapeutic target for PCa. Our study systematically reveals, for the first time, the critical role of disulfidptosis in PCa prognosis and immune modulation. Our key innovations include: A disulfidptosis subtyping framework identifying Cluster B with enhanced immune infiltration; A Dis score model prognosticating PCa survival and immunotherapy response; Experimental validation of CCNB2 as a disulfidptosis regulator via dual-pathway activation. These findings address the clinical knowledge gap in disulfidptosis and reposition CCNB2 beyond its classical cell-cycle role.

## Conclusion

Our study suggested that disulfidptosis was pivotal in assessing the prognosis of PCa. We have examined CCNB2's role in PCa progression and its underlying mechanisms, potentially paving the way for a novel therapeutic strategy for PCa treatment.

## Supplementary Material

Supplementary figures and tables.

## References

[1] Pinsky PF, Miller E, Prorok P, Grubb R, Crawford ED, Andriole G (2019). Extended follow-up for prostate cancer incidence and mortality among participants in the Prostate, Lung, Colorectal and Ovarian randomized cancer screening trial. BJU Int.

[2] Han J, Zhang J, Zhang W, Zhang D, Li Y, Zhang J (2019). Abiraterone and MDV3100 inhibits the proliferation and promotes the apoptosis of prostate cancer cells through mitophagy. Cancer Cell Int.

[3] Mulati Y, Lai C, Luo J, Hu J, Xu X, Kong D (2024). Establishment of a prognostic risk prediction model incorporating disulfidptosis-related lncRNA for patients with prostate cancer. BMC Cancer.

[4] Liu X, Nie L, Zhang Y, Yan Y, Wang C, Colic M (2023). Actin cytoskeleton vulnerability to disulfide stress mediates disulfidptosis. Nat Cell Biol.

[5] Tsvetkov P, Coy S, Petrova B, Dreishpoon M, Verma A, Abdusamad M (2022). Copper induces cell death by targeting lipoylated TCA cycle proteins. Science.

[6] Wang X, Zheng C, Yao H, Guo Y, Wang Y, He G (2024). Disulfidptosis: Six Riddles Necessitating Solutions. Int J Biol Sci.

[7] Peng X, Pan K, Zhao W, Zhang J, Yuan S, Wen X (2018). NPTX1 inhibits colon cancer cell proliferation through down-regulating cyclin A2 and CDK2 expression. Cell Biol Int.

[8] Kanska J, Zakhour M, Taylor-Harding B, Karlan BY, Wiedemeyer WR (2016). Cyclin E as a potential therapeutic target in high grade serous ovarian cancer. Gynecol Oncol.

[9] Kuner R, Fälth M, Pressinotti NC, Brase JC, Puig SB, Metzger J (2013). The maternal embryonic leucine zipper kinase (MELK) is upregulated in high-grade prostate cancer. J Mol Med (Berl).

[10] De Martino I, Visone R, Wierinckx A, Palmieri D, Ferraro A, Cappabianca P (2009). HMGA proteins up-regulate CCNB2 gene in mouse and human pituitary adenomas. Cancer Res.

[11] Zhou H, Wang L, Huang J, Jiang M, Zhang X, Zhang L (2015). High EGFR_1 Inside-Out Activated Inflammation-Induced Motility through SLC2A1-CCNB2-HMMR-KIF11-NUSAP1-PRC1-UBE2C. J Cancer.

[12] Mao P, Bao G, Wang YC, Du CW, Yu X, Guo XY (2020). PDZ-Binding Kinase-Dependent Transcriptional Regulation of CCNB2 Promotes Tumorigenesis and Radio-Resistance in Glioblastoma. Transl Oncol.

[13] Li R, Jiang X, Zhang Y, Wang S, Chen X, Yu X (2019). Cyclin B2 Overexpression in Human Hepatocellular Carcinoma is Associated with Poor Prognosis. Arch Med Res.

[14] Lei CY, Wang W, Zhu YT, Fang WY, Tan WL (2016). The decrease of cyclin B2 expression inhibits invasion and metastasis of bladder cancer. Urol Oncol.

[15] Park SH, Yu GR, Kim WH, Moon WS, Kim JH, Kim DG (2007). NF-Y-dependent cyclin B2 expression in colorectal adenocarcinoma. Clin Cancer Res.

[16] Gao CL, Wang GW, Yang GQ, Yang H, Zhuang L (2018). Karyopherin subunit-α 2 expression accelerates cell cycle progression by upregulating CCNB2 and CDK1 in hepatocellular carcinoma. Oncol Lett.

[17] Cai F, Li J, Zhang J, Huang S (2022). Knockdown of Circ_CCNB2 Sensitizes Prostate Cancer to Radiation Through Repressing Autophagy by the miR-30b-5p/KIF18A Axis. Cancer Biother Radiopharm.

[18] Liu Z, Sun L, Zhu W, Zhu J, Wu C, Peng X (2024). Disulfidptosis signature predicts immune microenvironment and prognosis of gastric cancer. Biol Direct.

[19] Ke S, Guo J, Wang Q, Shao H, He M, Li T (2023). Netrin Family Genes as Prognostic Markers and Therapeutic Targets for Clear Cell Renal Cell Carcinoma: Netrin-4 Acts through the Wnt/β-Catenin Signaling Pathway. Cancers (Basel).

[20] He L, Ioannidis A, Hoffman CJ, Arambula E, Joshi P, Whitelegge J (2024). Activation of the Mevalonate Pathway in Response to Anti-cancer Treatments Drives Glioblastoma Recurrences Through Activation of Rac-1. Cancer Res Commun.

[21] Ke S, Liu Z, Wang Q, Zhai G, Shao H, Yu X (2022). FAM107A Inactivation Associated with Promoter Methylation Affects Prostate Cancer Progression through the FAK/PI3K/AKT Pathway. Cancers (Basel).

[22] Li W, Jiang WS, Su YR, Tu KW, Zou L, Liao CR (2023). PINK1/Parkin-mediated mitophagy inhibits osteoblast apoptosis induced by advanced oxidation protein products. Cell Death Dis.

[23] Li J, Hong Z, Zhang J, Zheng S, Wan F, Liu Z (2024). Lysine methyltransferase SMYD2 enhances androgen receptor signaling to modulate CRPC cell resistance to enzalutamide. Oncogene.

[24] Chen H, Yang W, Xue X, Li Y, Jin Z, Ji Z (2022). Integrated Analysis Revealed an Inflammatory Cancer-Associated Fibroblast-Based Subtypes with Promising Implications in Predicting the Prognosis and Immunotherapeutic Response of Bladder Cancer Patients. Int J Mol Sci.

[25] Yan M, Wang J, Wang H, Zhou J, Qi H, Naji Y (2023). Knockdown of NR3C1 inhibits the proliferation and migration of clear cell renal cell carcinoma through activating endoplasmic reticulum stress-mitophagy. J Transl Med.

[26] Qian X, Song X, He Y, Yang Z, Sun T, Wang J (2015). CCNB2 overexpression is a poor prognostic biomarker in Chinese NSCLC patients. Biomed Pharmacother.

[27] Mo ML, Chen Z, Li J, Li HL, Sheng Q, Ma HY (2010). Use of serum circulating CCNB2 in cancer surveillance. Int J Biol Markers.

[28] Ni KW, Sun GZ (2019). The identification of key biomarkers in patients with lung adenocarcinoma based on bioinformatics. Math Biosci Eng.

[29] Zheng T, Liu Q, Xing F, Zeng C, Wang W (2023). Disulfidptosis: a new form of programmed cell death. J Exp Clin Cancer Res.

[30] Yu J, Raia P, Ghent CM, Raisch T, Sadian Y, Cavadini S (2021). Structural basis of human separase regulation by securin and CDK1-cyclin B1. Nature.

[31] Bouftas N, Wassmann K (2019). Cycling through mammalian meiosis: B-type cyclins in oocytes. Cell Cycle.

[32] Fang Y, Yu H, Liang X, Xu J, Cai X (2014). Chk1-induced CCNB1 overexpression promotes cell proliferation and tumor growth in human colorectal cancer. Cancer Biol Ther.

[33] Wu T, Zhang X, Huang X, Yang Y, Hua X (2010). Regulation of cyclin B2 expression and cell cycle G2/m transition by menin. J Biol Chem.

