# Supplementary figures and images for: Disulfidptosis-Associated CCNB2: A Prognostic Biomarker and Immune Microenvironment Modulator in Prostate Cancer
Source: J Cancer. 2025 Sep 3;16(13):3928–41. doi: 10.7150/jca.112791 (PMC12491202; doi:10.7150/jca.112791)

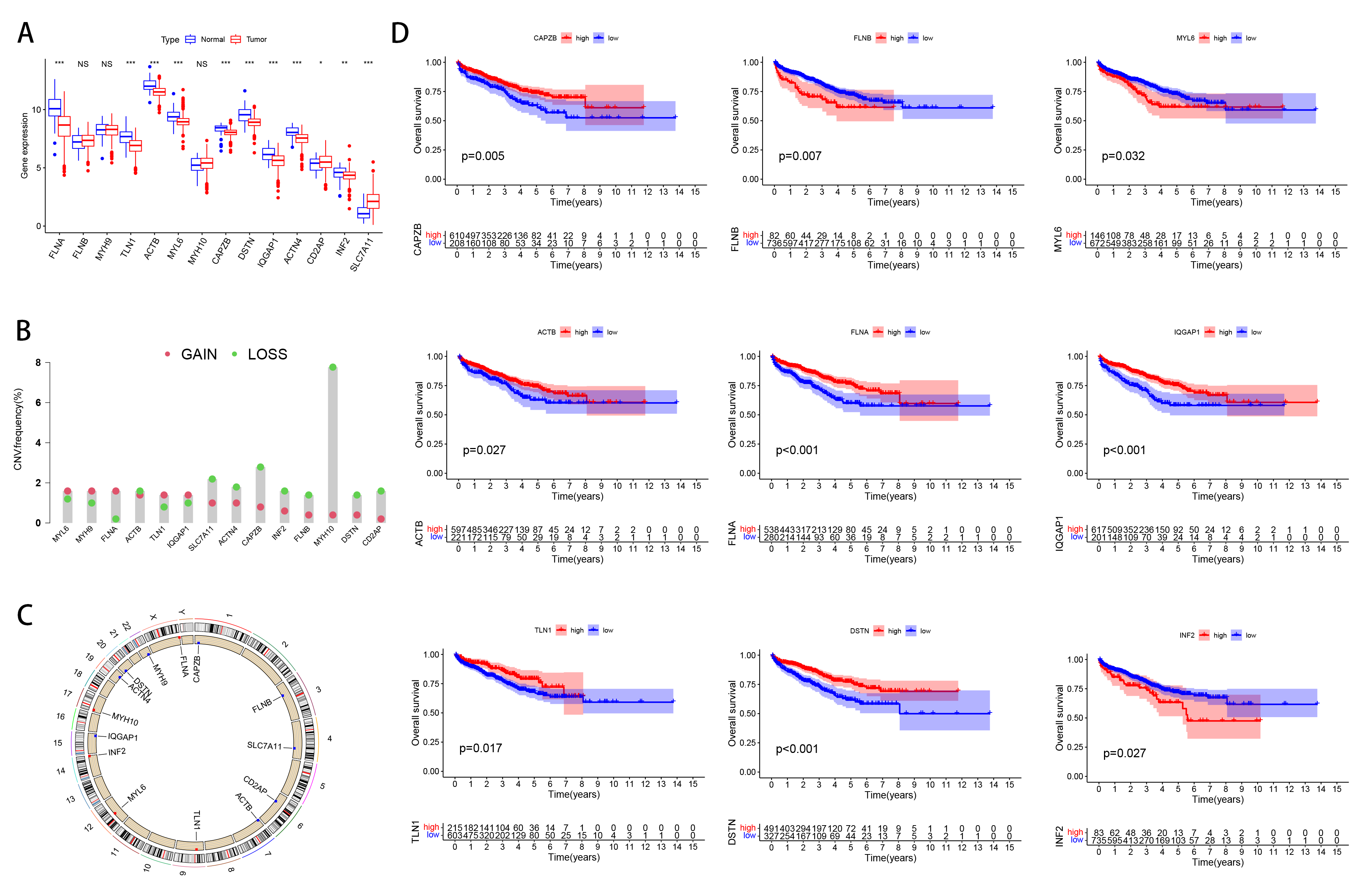

Supplement: Supplementary file 1 — Supplementary figures and tables. [file jcav16p3928s1.zip › Supply Tables and Figures/Figure1.tif]

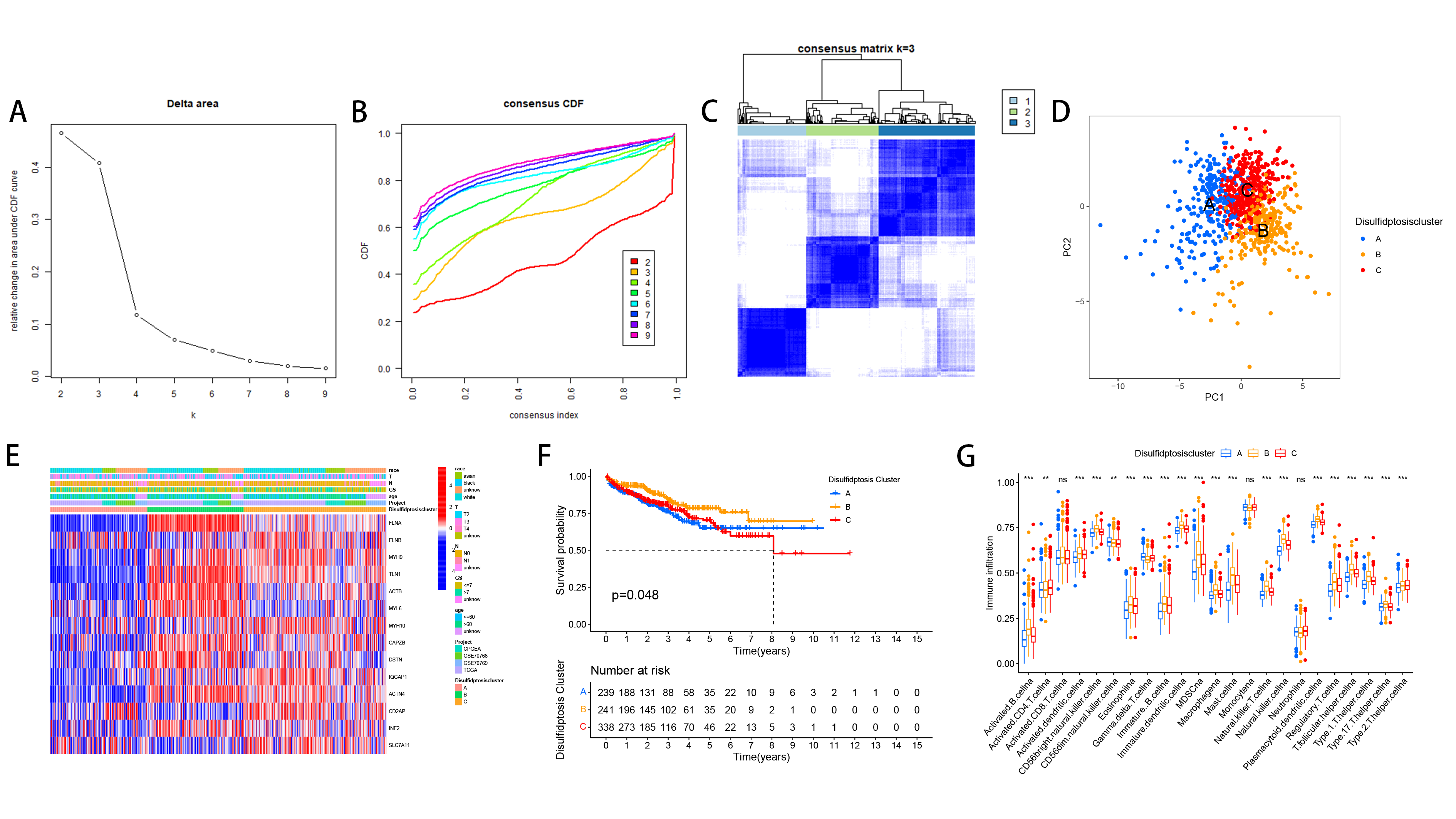

Supplement: Supplementary file 1 — Supplementary figures and tables. [file jcav16p3928s1.zip › Supply Tables and Figures/Figure2.tif]

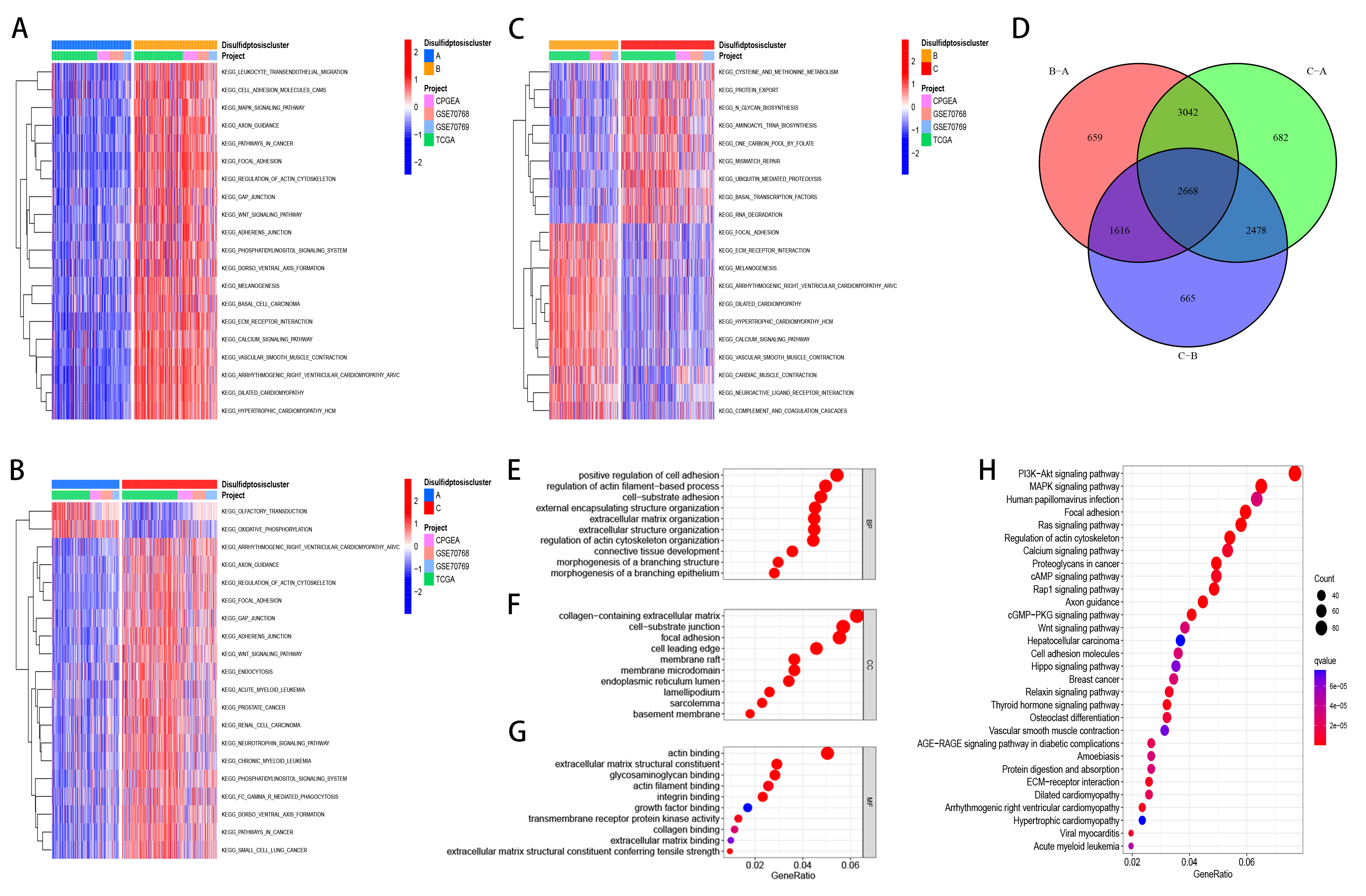

Supplement: Supplementary file 1 — Supplementary figures and tables. [file jcav16p3928s1.zip › Supply Tables and Figures/Figure3.tif]

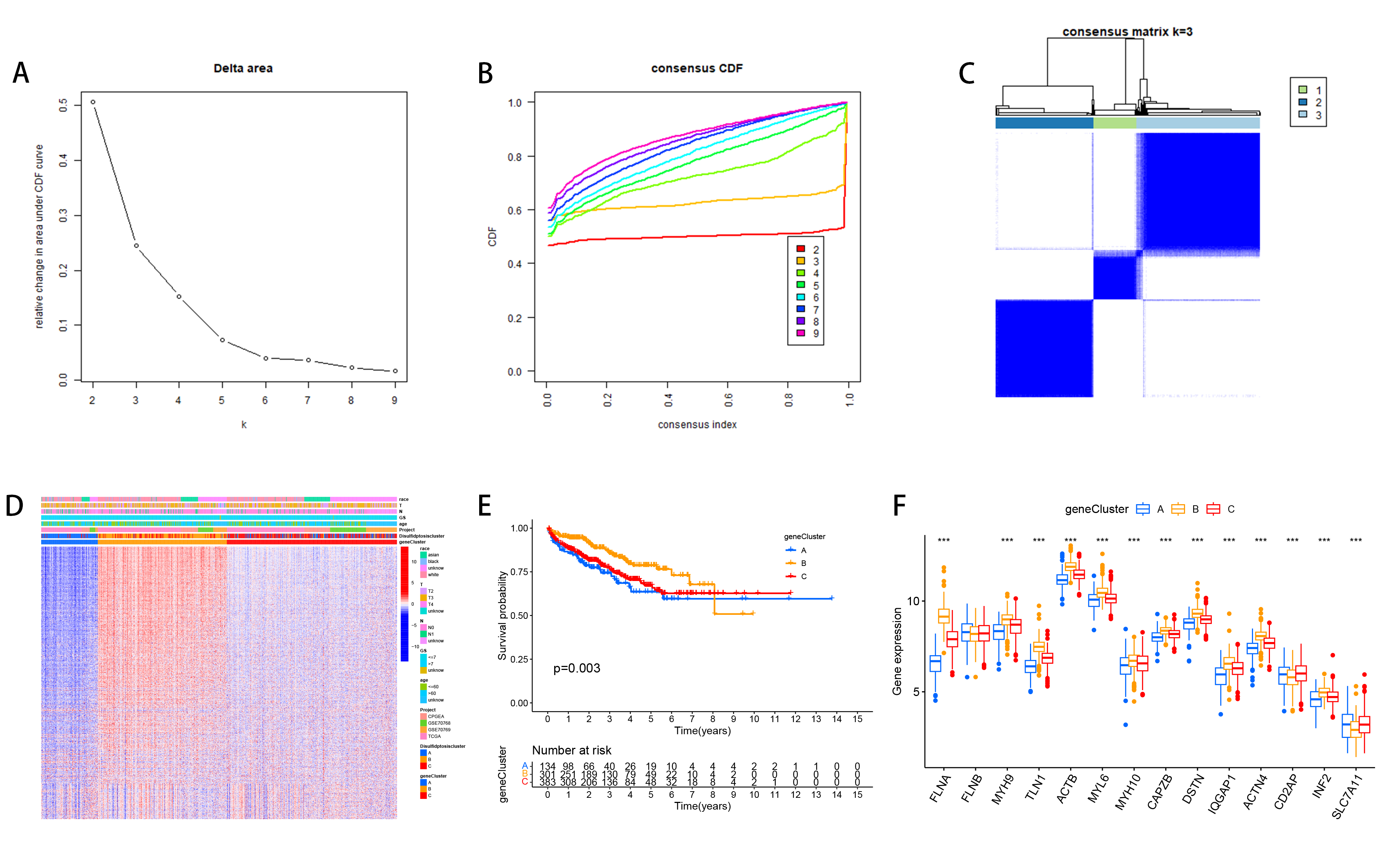

Supplement: Supplementary file 1 — Supplementary figures and tables. [file jcav16p3928s1.zip › Supply Tables and Figures/Figure4.tif]

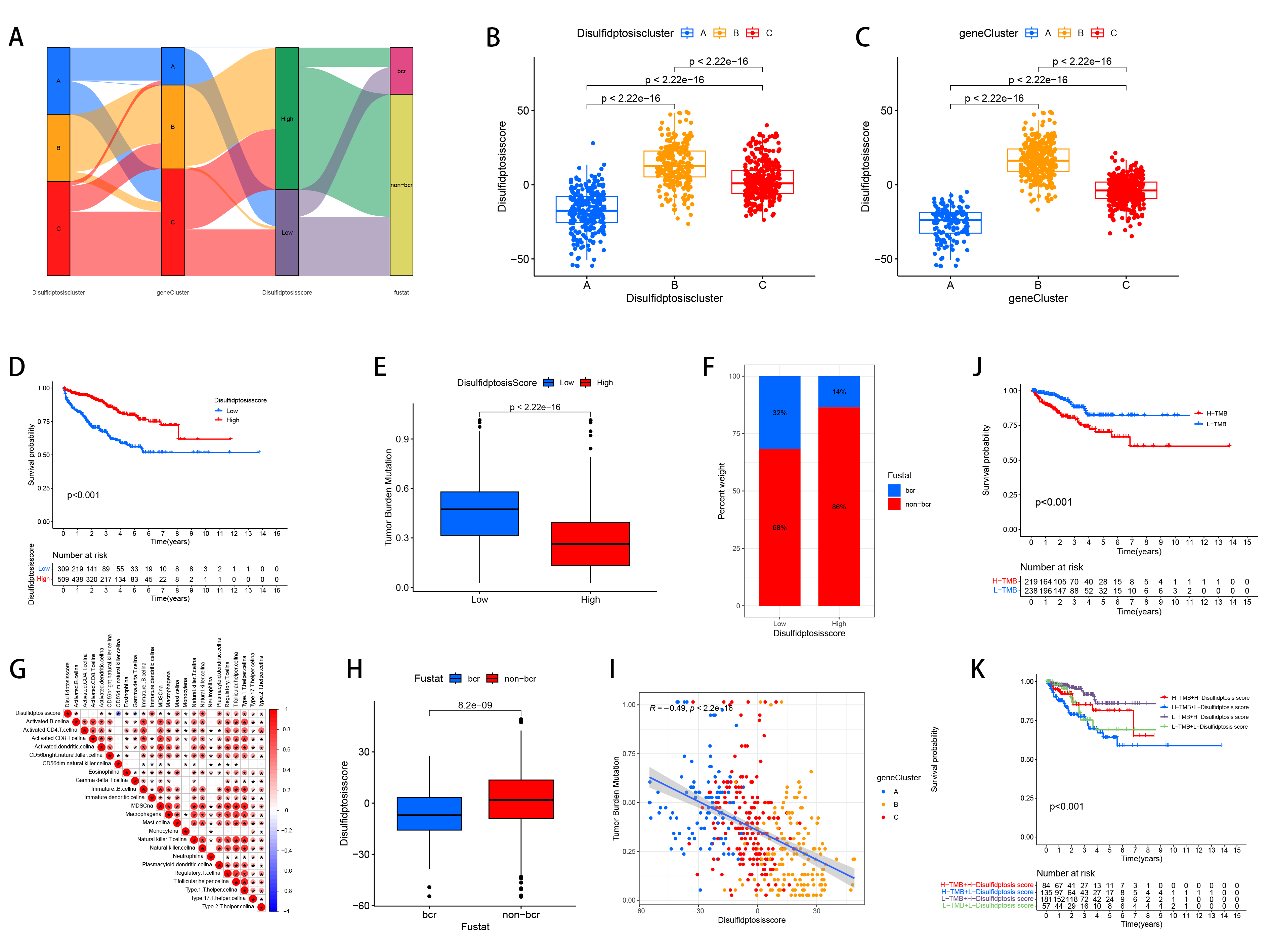

Supplement: Supplementary file 1 — Supplementary figures and tables. [file jcav16p3928s1.zip › Supply Tables and Figures/Figure5.tif]

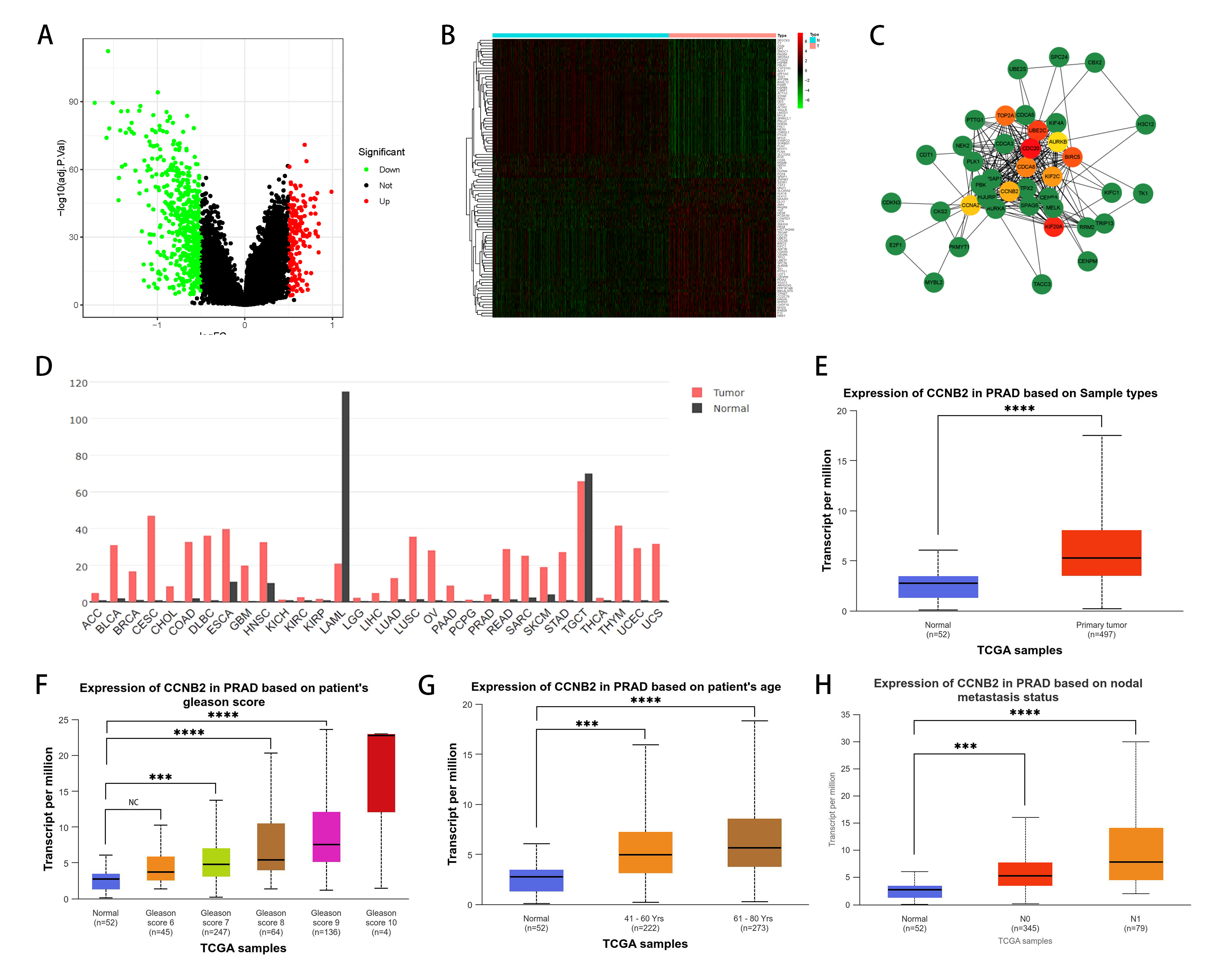

Supplement: Supplementary file 1 — Supplementary figures and tables. [file jcav16p3928s1.zip › Supply Tables and Figures/Figure6.tif]

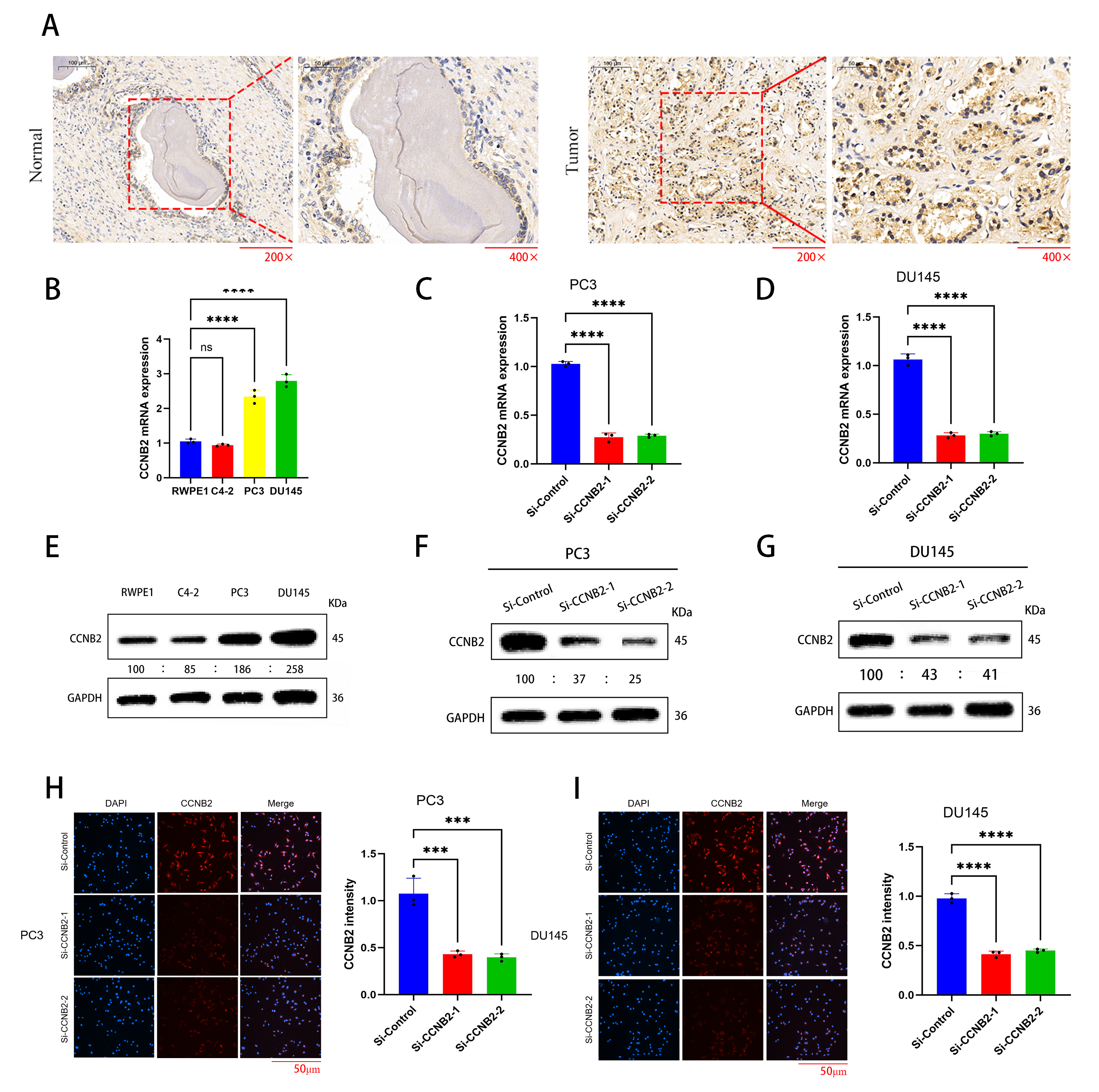

Supplement: Supplementary file 1 — Supplementary figures and tables. [file jcav16p3928s1.zip › Supply Tables and Figures/Figure7.tif]

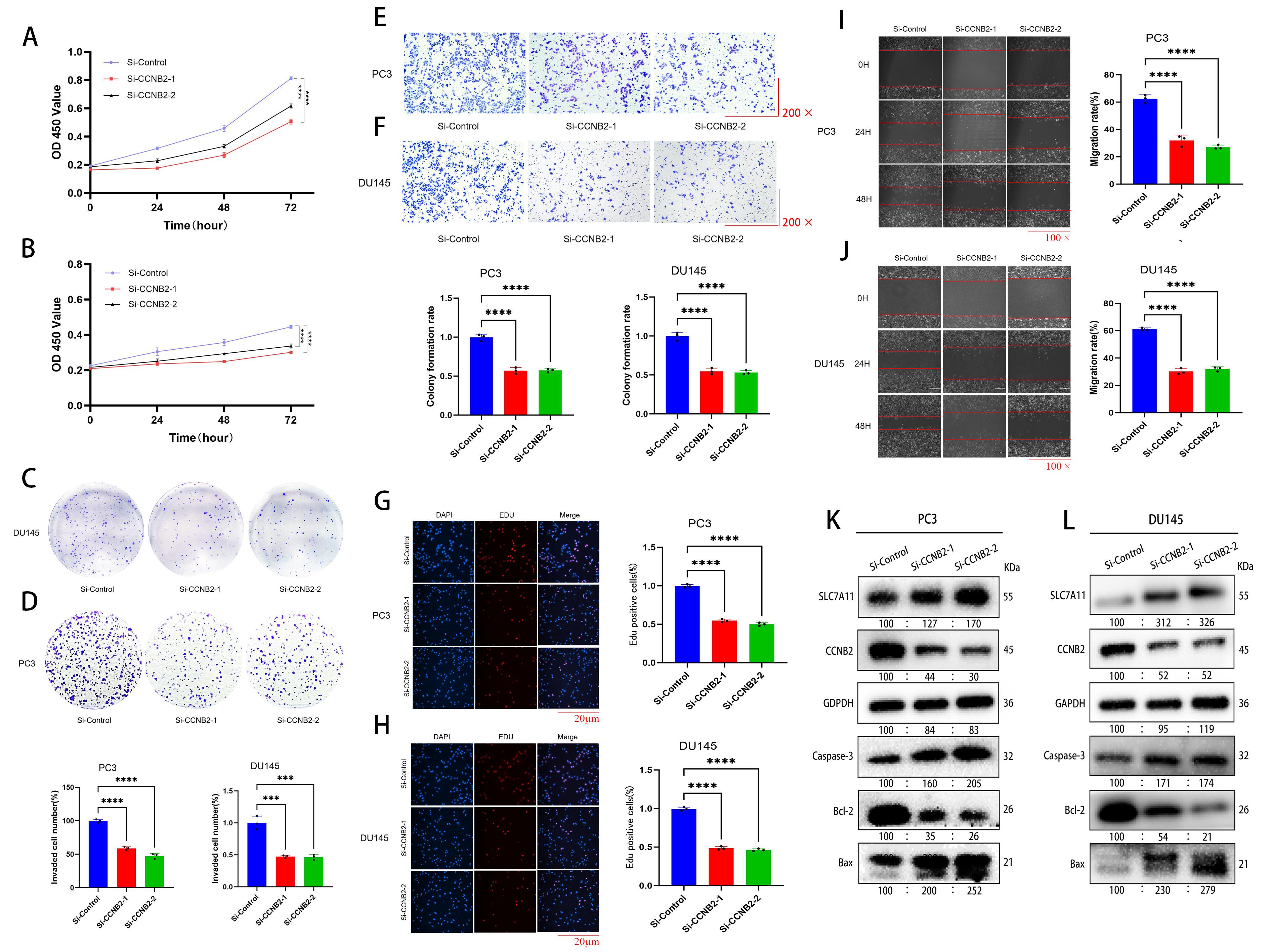

Supplement: Supplementary file 1 — Supplementary figures and tables. [file jcav16p3928s1.zip › Supply Tables and Figures/Figure8.tif]

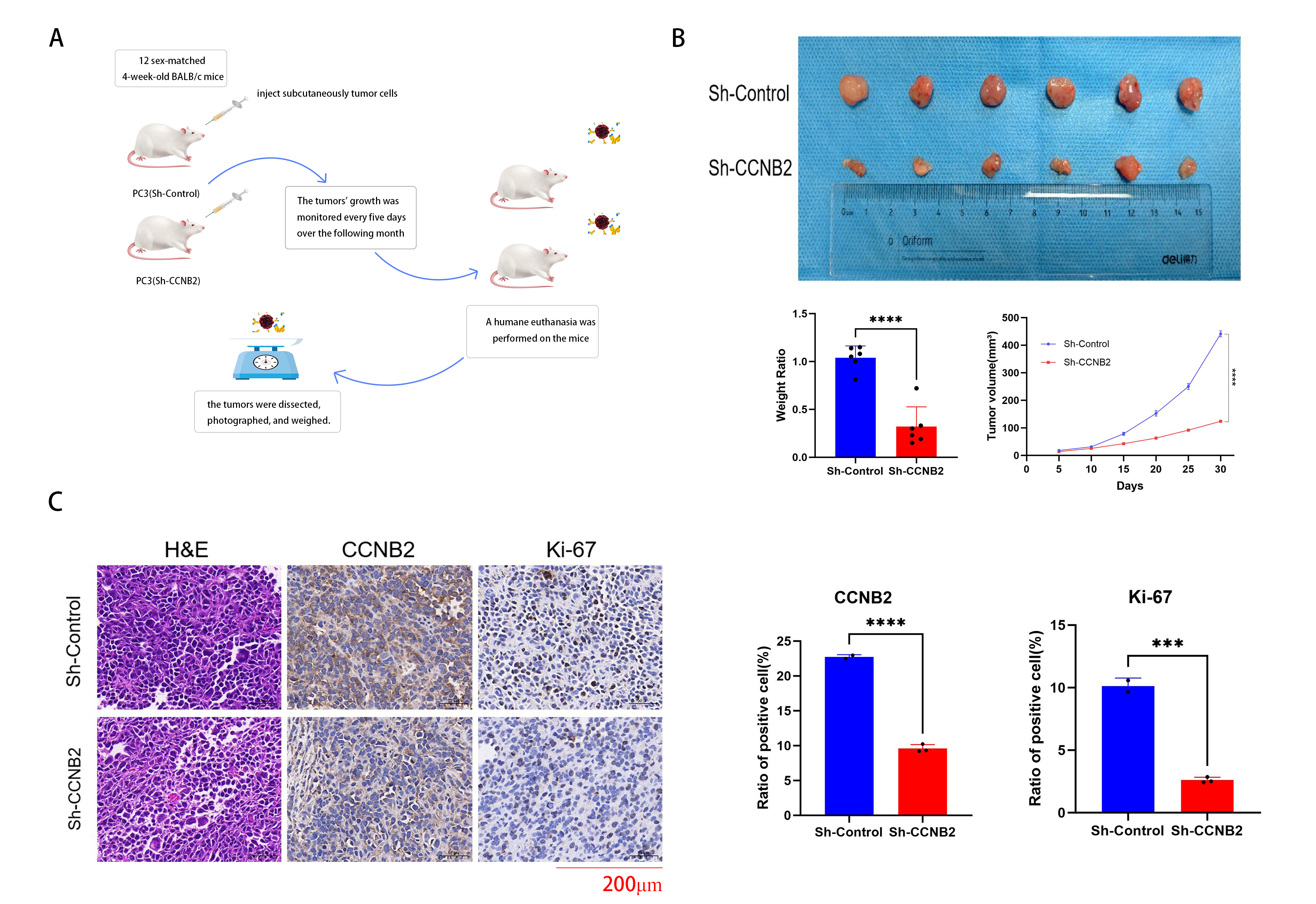

Supplement: Supplementary file 1 — Supplementary figures and tables. [file jcav16p3928s1.zip › Supply Tables and Figures/Figure9.tif]
